# SREBP1c modulates Treg immunobiology through a phospholipid-dependent adenosine pathway

**DOI:** 10.1172/JCI201325

**Published:** 2026-07-21

**Authors:** Fabrizia Bonacina, Claudio Procaccini, Marta Iaia, Arianna Moretti, Monika Svecla, Silvia Pedretti, Jeroen Bogie, Giovanni Battista Vingiani, Annalisa Moregola, Francesca Genova, Claudia Russo, Giusy De Rosa, Claudia La Rocca, Giada Mondanelli, Marco Gargaro, Nico Mitro, Giuseppe Matarese, Giuseppe Danilo Norata

**Affiliations:** 1 Department of Excellence of Pharmacological and Biomolecular Sciences “Rodolfo Paoletti,” Università degli Studi di Milano, Milan, Italy.; 2 Laboratorio di Immunologia, Istituto degli Endotipi in Oncologia, Metabolismo e Immunologia “G. Salvatore” – Consiglio Nazionale delle Ricerche, Napoli, Italy.; 3 Unità di Neuroimmunologia, IRCCS Fondazione Santa Lucia, Roma, Italy.; 4 Deutsches Herzzentrum der Charité – Charité-Universitätsmedizin, Berlin, Germany.; 5 Department of Experimental Oncology, European Institute of Oncology (IEO) IRCCS, Milan, Italy.; 6 Department of Immunology and Infection, Hasselt University, Diepenbeek, Belgium.; 7 University MS Center, Pelt, Belgium.; 8 Dipartimento Assistenziale Integrato de Medicina di Laboratorio e Trasfusionale, Azienda Ospedaliera Universitaria “Federico II,” Napoli, Italy.; 9 Department of Pharmaceutical Sciences, University of Perugia, Perugia, Italy.; 10 Treg Cell Lab, Dipartimento di Medicina Molecolare e Biotecnologie Mediche, Università degli Studi di Napoli “Federico II,” Napoli, Italy.

**Keywords:** Immunology, Metabolism, Lipidomics, Metabolomics, Tregs

## Abstract

Tregs maintain immune tolerance through mechanisms tightly coupled to cellular metabolism. Whereas glycolysis supports migration of Tregs, lipid metabolism sustains their suppressive phenotype. Here, we identify SREBP1c as a central regulator of Treg immunobiology. Tregs from *Srebp1c*-deficient mice displayed impaired suppressive function, reduced frequencies in circulation and lymphoid tissues, and diminished expression of functional markers. These defects stemmed from intrinsic metabolic rewiring rather than systemic alterations, as both ex vivo Tregs (CD4^+^CD25^hi^FoxP3^+^) and in vitro–derived Tregs lacking *Srebp1c* were shifted toward glycolysis. Integrated transcriptomic and lipidomic analyses revealed that *Srebp1c*-deficient Tregs exhibited defective phospholipid remodeling, with an accumulation of lysophosphatidylcholines over phosphatidylcholines, which we attributed to enhanced cytosolic phospholipase A2 (cPLA2α) activity and disruption of the Lands cycle. Altered lipid composition impaired adenosine-mediated immunosuppression by reducing CD73 expression and extracellular adenosine generation. Accordingly, pharmacological inhibition of cPLA2α restored adenosine signaling, CD73 expression, and Treg suppressive capacity. Thus, by preserving phospholipid homeostasis, SREBP1c functions as an immunometabolic checkpoint that links lipid metabolism to adenosine-dependent Treg suppression.

## Abstract 2

A key regulator of intracellular lipid balance, SREBP1c, controls the tolerogenic activity of regulatory T cells by modulating their function and metabolism.

## Graphical abstract

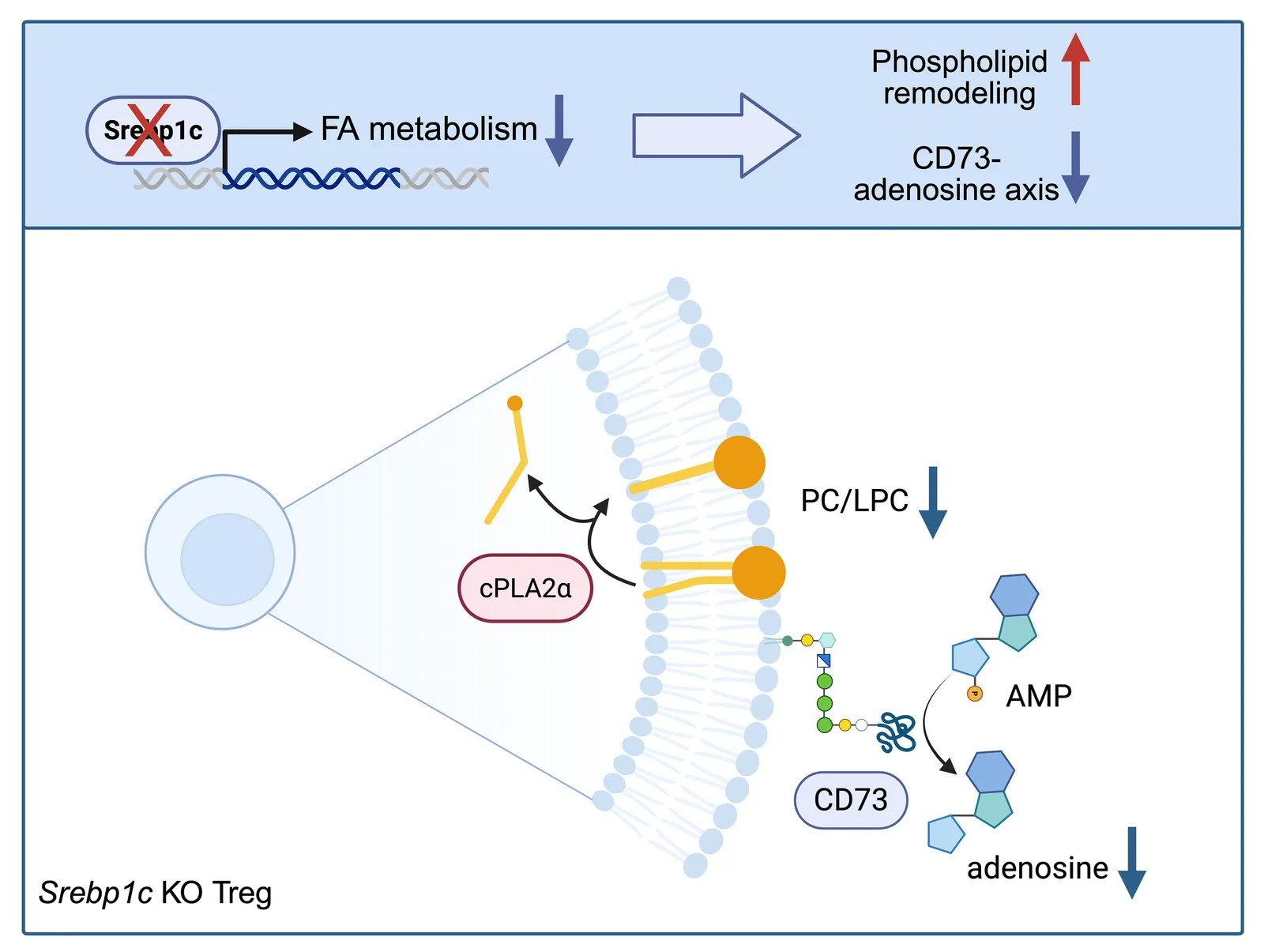

## Introduction

Tregs are a subset of CD4^+^ T lymphocytes that maintain immune surveillance and *self-*tolerance (1). Their function is closely related to cellular metabolism, with immunosuppressive activity generally associated with increased oxidative metabolism, whereas cell growth and migration rely on glycolysis (2–4). FOXP3, the main Treg transcription factor (TF), restrains glycolysis and unleashes fatty acid (FA) metabolism, supporting Treg survival and suppressive function (5, 6). This balance is influenced by the microenvironment, with Treg metabolic profiles varying between in vitro and ex vivo settings. These contrasting findings suggest dynamic, tissue-specific regulation and highlight uncertainties regarding the role of lipid modulation in Treg function.

Lipid homeostasis is coordinated by the SREBPs, a family of basic helix loop helix leucine zipper (bHLH-LZ) TFs, which are encoded by two genes (*Srebp1* and *Srebp2*) responsible for both cholesterol and FA metabolism (7). While SREBP1a (one of the two isoforms coded from the *Srebp1* gene) and SREBP2 mainly control the transcription of genes involved in cholesterol synthesis and uptake, SREBP1c controls the transcription of genes required for FA synthesis and metabolism. This difference is reflected in the metabolic cues that drive activation of the different TFs in the liver: SREBP1a and SREBP2 are activated by intracellular sterol deprivation, while SREBP1c is activated in response to nutrition (insulin/LXR activation) (8). This mechanism has been broadly studied in metabolic tissues, such as in hepatocytes, where SREBP1c activation promotes the conversion of acetyl-CoA derived from glycolysis into substrates for FA synthesis (9). Nevertheless, several nonlipogenic genes have been shown to be regulated in a cell-specific manner by SREBP1c (10), extending the role of this TF beyond that of a mere gatekeeper of lipid energy metabolism.

Building on this premise, we investigated whether SREBP1c could serve as a novel immunometabolic checkpoint regulating Treg immunobiology. To this aim, we profiled Tregs from *Srebp1c*-KO mice via a combination of metabolomics, lipidomics, and transcriptomics, showing that *Srebp1c* deficiency impairs Treg immunosuppressive functions. In addition to a rewiring of Treg metabolism toward glycolysis, we unveiled that FA chain remodeling of phospholipids (PL), a process known as Lands cycle that is involved in receptor clustering at the plasma membrane and transduction of intracellular signalling, was impaired in *Srebp1c*-KO Tregs. Specifically, we reported an accumulation of lysophosphatidylcholines (LPC) over phosphatidylcholines (PC) and a compromised adenosine pathway in *Srebp1c-*KO Tregs. Thus, we uncovered the role of SREBP1c in controlling PL composition and Treg suppressive function by modulating adenosine pathway and energy metabolism, promoting FA metabolism and limiting glucose catabolism. Furthermore, we identified cytosolic phospholipase A2 (cPLA2α), a key enzyme of the Lands cycle, as the driver of the Treg phenotype and a targetable mediator of adenosine-mediated immunosuppression in Tregs.

## Results

### Srebp1c deficiency worsens Treg immunosuppressive phenotype.

**Figure 1:**
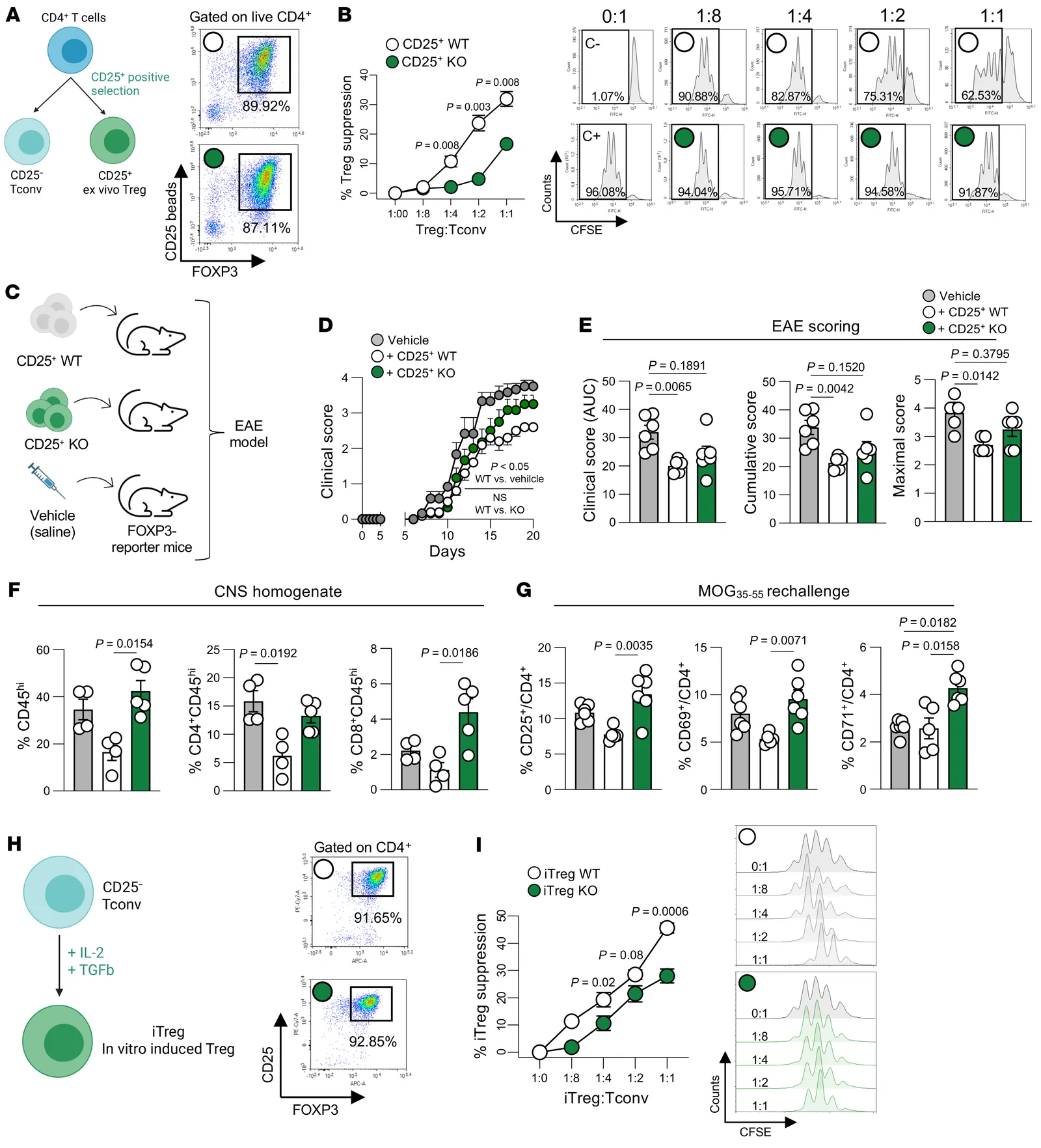
Tregs from *Srebp1c*-KO mice showed an impaired suppressive function. (**A**) Graphic representation of ex vivo Tregs purified as CD25^+^ from CD4^+^ T cells; a representative flow cytometry dot plot of live isolated cells expressing CD4/CD25/FOXP3 is shown. (**B**) Suppression of CD4^+^CD25^–^ (Tconv) proliferation by freshly isolated Tregs (CD25^+^) from lymphoid tissues of *Srebp1c*-KO and WT mice cultured at different ratios (0:1 indicates that no Tregs were used, while 1:8, 1:4 1:2, and 1:1 indicate the proportion of Tregs to Tconv) for 4 days. Representative histograms from flow cytometry are shown. (**C**) Graphic representation of the EAE protocol: FOXP3-reporter (B6.Cg-Foxp3^tm2Tch^/J) mice received purified WT or *Srebp1c*-KO Tregs, or saline as control (vehicle), and thereafter were immunized with MOG and pertussis toxin. (**D** and **E**) Daily recorded EAE clinical score (**D**) and relative parameters (**E**) AUC of clinical, maximal, and cumulative scores) of EAE mice treated as indicated in **D**. (**F**) Immune infiltrate of total leukocytes (CD45^hi^) and T cell subsets (CD4 and CD8) in the CNS of EAE mice treated as indicated in **D**, 20 days after MOG_35–55_ immunization. (**G**) Expression of activation markers in CD4^+^ T cells in splenocytes isolated from EAE mice and in vitro rechallenged with MOG_35–55_ peptide 20 days after MOG_35–55_ immunization. (**H**) In vitro–induced Tregs (iTregs) were differentiated from CD4^+^CD25^–^ cells by culture with IL-2 (100 U/mL) and TGF-β (5 ng/mL) for 4 days. Representative dot plots of CD25 and FOXP3 expression from flow cytometry of iTregs are shown. (**I**) Suppression of Tconv proliferation by WT and *Srebp1c*-KO iTregs for 3 days. Representative histograms from flow cytometry are shown. *N* = 4–10/group. Data are presented as mean ± SEM. Statistical analysis has been performed with multiple Mann-Whitney tests (**B** and **I**), 2-way ANOVA multiple comparisons (**D**), and 1-way ANOVA Kruskal-Wallis test (**E**–**G**).

To investigate the role of SREBP1c in Treg biology, we purified CD4^+^CD25^+^ cells from the spleens of *Srebp1c* WT and -KO mice, which were confirmed to be Tregs by FoxP3 expression (Figure 1A), to perform in vitro and in vivo functional assays. Ex vivo–isolated CD4^+^CD25^+^ Tregs from *Srebp1c*-KO mice, when cocultured at increasing ratios with CD4^+^CD25^–^ conventional T cells (Tconv), were less efficient in suppressing Tconv proliferation as compared with those from WT mice (Figure 1B), suggesting a functional impairment in Treg suppressive potency in the absence of SREBP1c. We further validated this dysfunctional phenotype by assessing the ability of purified CD25^+^ cells (Tregs) to ameliorate the severity of experimental autoimmune encephalomyelitis (EAE), an autoimmune-mediated demyelinating disease of the CNS. Tregs isolated from either WT or KO mice were injected into C57BL/6J-FOXP3 reporter recipients 16 hours prior to EAE induction. Disease progression was monitored compared with that in mice receiving vehicle (saline) (Figure 1C). The administration of WT Tregs was able to significantly attenuate EAE progression (Figure 1D), as evidenced by a marked reduction in both the maximal and cumulative EAE disease scores compared with the vehicle control group (Figure 1E). Conversely, mice that received *Srebp1c*-KO Tregs exhibited disease scores that were not different from those of the saline group (Figure 1, D and E), thus indicating that, unlike WT Tregs, *Srebp1c*-deficient Tregs do not significantly ameliorate EAE severity. Consistent with these findings, the immunoinflammatory infiltrate was reduced in the CNS of EAE mice treated with WT Tregs, as suggested by the decreased frequency of CD45^+^ leukocytes (including both CD4^+^ and CD8^+^ T cells), but not in those treated with *Srebp1c*-KO Tregs (Figure 1F). This effect was paralleled by a similar frequency of recipient Tregs (identified as GFP^+^ cells) in the spleens of mice treated with *Srebp1c*-KO Tregs and those receiving saline, whereas a higher frequency was observed in mice receiving WT Tregs (Supplemental Figure 1A; supplemental material available online with this article; https://doi.org/10.1172/JCI201325DS1), suggesting the establishment of a tolerogenic environment. In line with this, the expression of Treg-associated suppressive markers (PD-1, Tigit, CTLA-4, CD62L, CD69, and CCR7) was positively regulated in WT Treg-treated mice, with PD-1 and CD62L reaching a statistically significant difference compared with mice receiving *Srebp1c*-KO Tregs (Supplemental Figure 1B). Furthermore, following in vitro cognate antigen-specific stimulation of splenocytes isolated from EAE mice, myelin oligodendrocyte glycoprotein peptide 35–55 (MOG_35–55_) reactive–CD4^+^ T cells from mice treated with *Srebp1c*-KO Tregs displayed higher expression of activation markers (CD25, CD69, and CD71) compared with those from mice receiving WT Tregs (Figure 1G). In parallel, polyclonal stimulation with anti-CD3 of splenocytes from EAE mice resulted in a significant reduction of OX-40 only in CD4^+^ T cells of mice that received WT Tregs compared with saline-treated controls (Supplemental Figure 1C). In contrast, mice receiving *Srebp1c*-KO Tregs, did not show this trend, with a superimposable expression of CD25, CD69, and OX-40 in activated CD4^+^ T cells compared with mice receiving saline, pointing to an impaired potency of *Srebp1c*-deficient Tregs in controlling systemic immune response upon EAE induction.

Given that *Srebp1c* hepatic deficiency reduces systemic triglyceride (TG) and cholesterol levels (11), we next sought to exclude the effect of systemic lipid metabolism on Treg phenotype. To this end, we investigated the phenotype of Tregs differentiated in vitro (iTregs) from that of conventional CD4^+^ T cells. CD4^+^CD25^–^ cells were isolated from the lymph nodes of *Srebp1c*-KO or WT mice and stimulated with anti-CD3/28 antibodies in the presence of a high dose of IL-2 and TGF-β (Figure 1H). This approach resulted in nearly 90% of viable CD4^+^ cells expressing both CD25 and FOXP3 in both WT and *Srebp1c*-KO cells (Figure 1H). Even under this experimental setting (thereby eliminating the contribution of systemic lipid metabolism), *Srebp1c-*KO iTregs exhibited reduced immunosuppressive function compared with that of WT iTregs, as demonstrated by their impaired ability to dampen cell proliferation at increasing iTreg-to-Tconv ratios in coculture assays (Figure 1I).

To further corroborate the impaired ability of *Srebp1c*-KO Tregs to counteract immunoinflammatory responses, we used the 2,4,6-trinitrobenzene sulfonic acid–induced (TNBS-induced) colitis model, a well-established model of Th1/Th17-driven transmural inflammation that recapitulates key features of human Crohn’s disease (12, 13). Disease severity was monitored by assessing body weight loss and inflammatory parameters in the colon. Intrarectal administration of TNBS induced significant weight loss compared with that of vehicle-treated control mice (Supplemental Figure 2, A and B). Adoptive transfer of WT Tregs (CD25^+^) partially attenuated weight reduction, with a significant improvement observed at days 2 and 3 after induction. In contrast, the weight-loss profiles of mice receiving *Srebp1c*-KO Tregs (CD25^+^) were more similar to those of untreated colitic mice (Supplemental Figure 2, A and B). These findings were supported by colon length shortening in the TNBS and TNBS + Treg-KO groups compared with the TNBS + Treg WT and vehicle groups (Supplemental Figure 2C) and by a substantial reduction in the production of proinflammatory cytokines, including IL-17A, IFN-γ, and IL-6, in mice receiving WT, but not KO, Tregs. (Supplemental Figure 2, D–F). Notably, no significant differences in IL-10 levels were detected among the experimental groups (Supplemental Figure 2G), suggesting that the observed effects may be primarily associated with modulation of proinflammatory cytokine production. Overall, these results support the observation that *Srebp1c* deficiency may impair the immunosuppressive function of Tregs, thereby reducing their protective capacity under immunoinflammatory challenges.

Taken together, these findings underlined a reduced suppressive potency under *Srebp1c* deficient conditions, suggesting a key role for this TF in controlling Treg function.

### Srebp1c deficiency results in an impaired Treg distribution and phenotype in vivo.

**Figure 2:**
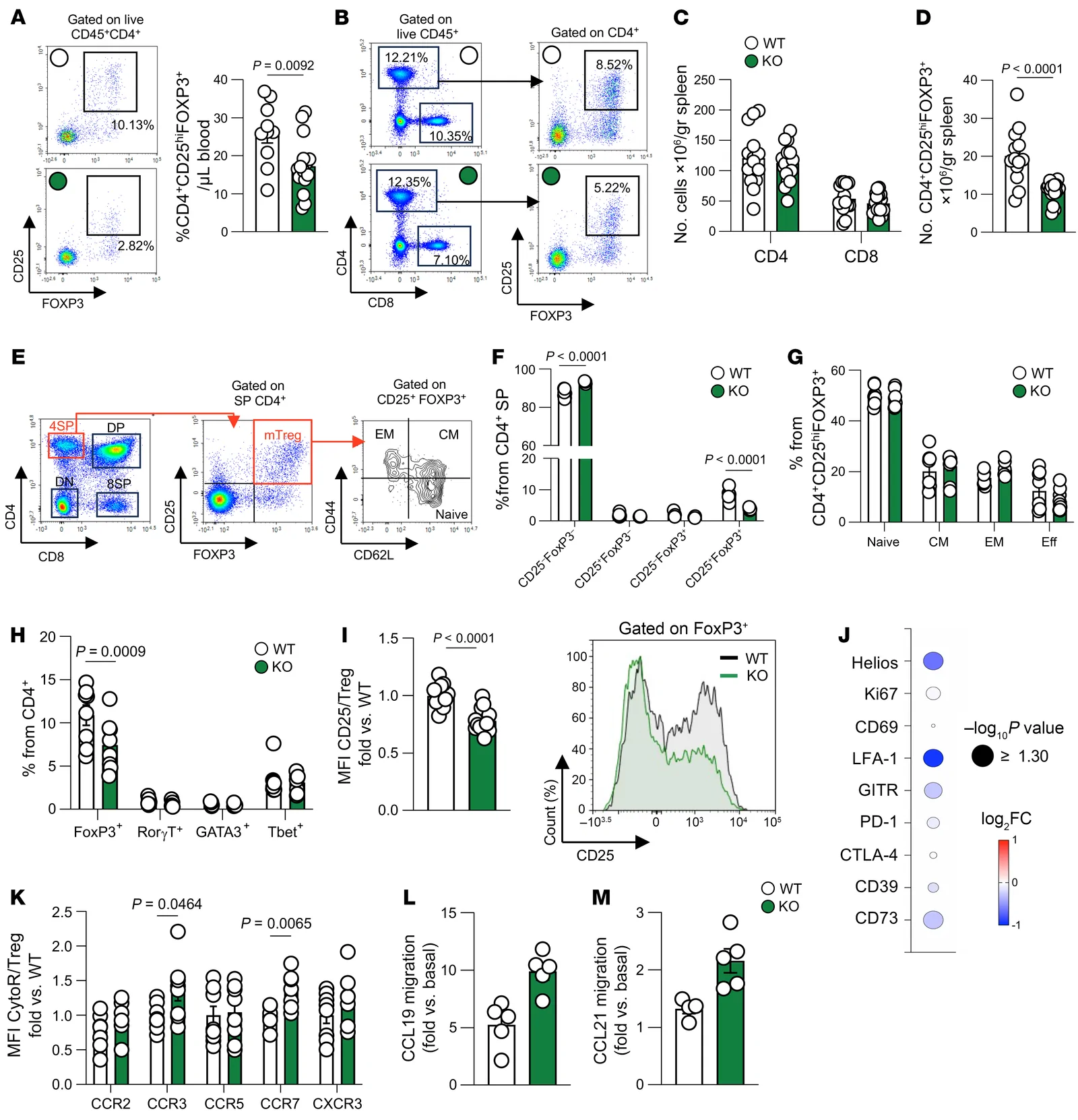
*Srebp1c* deficiency results in an impaired Treg distribution and phenotype. (**A**) Representative plots from flow cytometry showing CD25 and FOXP3 expression on CD4^+^ T cells in circulation and absolute count of Tregs (CD25^hi^FOXP3^+^). (**B**) Representative plots from flow cytometry showing CD4 and CD8 expression from total live leukocytes (CD45) and CD25 and FOXP3 on CD4^+^ T cells. (**C** and **D**) Numbers of CD4^+^ and CD8^+^ T cells (**C**) and Tregs (**D**) corrected for spleen weights. (**E**) Gating strategy used to identify Treg subsets in the thymus. (**F**) Frequency of thymic precursors and mature Tregs depending on the expression of CD25 and FOXP3. (**G**) Frequency of thymic naive (CD62L^+^CD44^–^), central memory (CD62L^+^CD44^+^) and effector memory (CD62L^–^CD44^+^) cells. (**H**) Frequency of FOXP3^+^, RORγT^+^, GATA3^+^, and T-BET^+^ in splenic CD4^+^ T cells. (**I**) Fold of expression of CD25 receptor within the splenic CD4^+^FOXP3^+^ population corrected to WT values; a representative histogram of CD25 expression within the FOXP3^+^ cells is shown. (**J**) Expression of markers of Treg stability (HELIOS), activation (KI67, CD69, LFA-1, GITR), and suppressive function (PD-1, CTLA-4, CD39, and CD73) in the spleens. Data are presented as log_2_ fold change (FC) of expression compared with WT: the size of the dot indicates the statistical significance, while colors indicate the log_2_FC (red for upregulated proteins, blue for downregulated proteins). (**K**) Expression of the chemokine receptors CCR2, CCR3, CCR5, CCR7, and CXCR3 in circulating Tregs. (**L** and **M**) In vitro migration assay of ex vivo–isolated Tregs (CD25^+^) toward CCL19 (**L**) and CXCL10 (**M**) chemokines after 3 hours of incubation in 24-well plate Transwells inserts. Results are corrected versus spontaneous (basal) migration toward the medium. Data are presented as mean ± SEM. *N* = 5–13/group. Statistical analysis has been performed with unpaired nonparametric *t* test (**A**, **D**, and **J**–**N**) or 2-way ANOVA (**C** and **F**–**H**).

To follow up on the functional characterization, we next investigated whether *Srebp1c* deficiency affected Treg distribution and phenotype under homeostatic conditions in vivo. Circulating levels of CD4^+^CD25^hi^FoxP3^+^ Tregs were markedly reduced in KO mice compared with WT littermates (Figure 2A and Supplemental Figure 3A). Similarly, while in the spleens the absolute counts of CD4^+^ and CD8^+^ T cells were not affected by *Srebp1c* deficiency (Figure 2, B and C, and Supplemental Figure 3B), CD4^+^CD25^hi^FoxP3^+^ Tregs were significantly decreased in KO compared with WT mice (Figure 2, B and D, and Supplemental Figure 3B). Notably, we observed a decreased frequency of both CD25^+^FoxP3^+^ and CD25^–^FoxP3^+^ Tregs in the spleens of *Srebp1c*-KO compared with WT mice (Supplemental Figure 3C). However, the distribution of naive and memory subsets was largely comparable between WT and KO mice (Supplemental Figure 3, D and E). To figure out whether reduced Treg levels resulted from an impaired thymic development, we analyzed Treg differentiation within single-positive CD4^+^ T cells tracking the expression of CD25, FOXP3, CD62L, and CD44 as markers of T cell maturation and distribution in secondary lymphoid organs (Figure 2E). In the thymi of *Srebp1c*-KO mice, we observed an increased frequency of the double-positive population (CD4^+^CD8^+^), and a slight reduction of single-positive CD4^+^ T cells (Supplemental Figure 3F), with no difference in the distribution between double-negative subpopulations (based on CD25 and CD44 expression; data not shown). Notably, *Srebp1c*-KO mice exhibited a significantly less prevalence of CD25^+^FOXP3^+^ cells and higher prevalence of CD25^–^FOXP3^–^ cells compared with WT mice, with no difference in the intermediate Treg subsets (CD25^–^FOXP3^+^ and CD25^+^FOXP3^–^) (Figure 2F). The comparable frequencies of naive, central, and effector memory subsets between KO and WT mature Tregs (Figure 2G) also excluded defects in Treg-antigen priming and trafficking between lymphoid organs and instead showed an impairment in the maturation to CD25^hi^FOXP3^+^ cells. Indeed, a comparable expression of the lineage-defining TFs RORγt, GATA3, and T-BET — associated with Th17, Th2, and Th1 subsets (14), respectively — was detected in peripheral CD4^+^ T cells from WT and *Srebp1c*-KO mice, whereas only the frequency of FOXP3^+^ cells was significantly reduced in *Srebp1c*-deficient CD4^+^ T cells (Figure 2H).

We excluded that the reduced frequency of CD4^+^CD25^hi^FOXP3^+^ Tregs resulted from a different tolerogenic polarization by myeloid cells, as no changes were observed in the frequencies of DCs and macrophages (Supplemental Figure 3G) or in the capacity of purified splenic CD11c^+^ cells to polarize allogeneic CD4^+^ T cells toward a specific T helper phenotype (Supplemental Figure 3H). This evidence further supported the notion that SREBP1c plays an intrinsic role in Treg biology. Furthermore, the expression of CD25, a key receptor protein involved in Treg suppressive function, was significantly reduced in *Srebp1c*-KO Tregs compared with WT cells (Figure 2I). This was paralleled by reduced expression of markers associated with Treg phenotype, such as HELIOS, PD-1, and CD73, with the first usually associated with Treg stability and the others with suppression potency (Figure 2J), overall indicating that SREBP1c plays a relevant role in maintaining the expression of typical Treg markers. Furthermore, circulating Tregs from *Srebp1c*-KO mice also showed increased expression of the chemokine receptors CCR3 and CCR7, along with a trend toward higher expression of CCR2 and CXCR3 (Figure 2K), suggesting altered migratory capacity of Tregs under *Srebp1c*-deficient conditions. Indeed, in an in vitro Transwell migration assay, ex vivo CD25^hi^FOXP3^+^ Tregs from *Srebp1c*-KO mice migrated more than WT cells toward both lymphoid-homing (CCL19) and inflammatory-homing (CXCL10) chemokines (Figure 2, L and M). Similar to ex vivo Tregs, iTregs from *Srebp1c-*KO mice showed increased migration toward both cytokines (CCL19 and CXCL10) compared with WT iTregs (Supplemental Figure 4, A and B). We have previously shown that Treg suppression versus migratory plasticity is mediated by immunometabolic reprogramming toward glycolysis (2). As such, a functional shift from suppression to migration under *Srebp1c*-deficient conditions could represent a possible compensatory mechanism, reflecting Treg plasticity. Taken together, these data highlighted the role of SREBP1c as a critical modulator of Treg-suppressive phenotype and function under physiological conditions.

### Srebp1c deficiency rewires Treg metabolism toward glycolysis.

**Figure 3:**
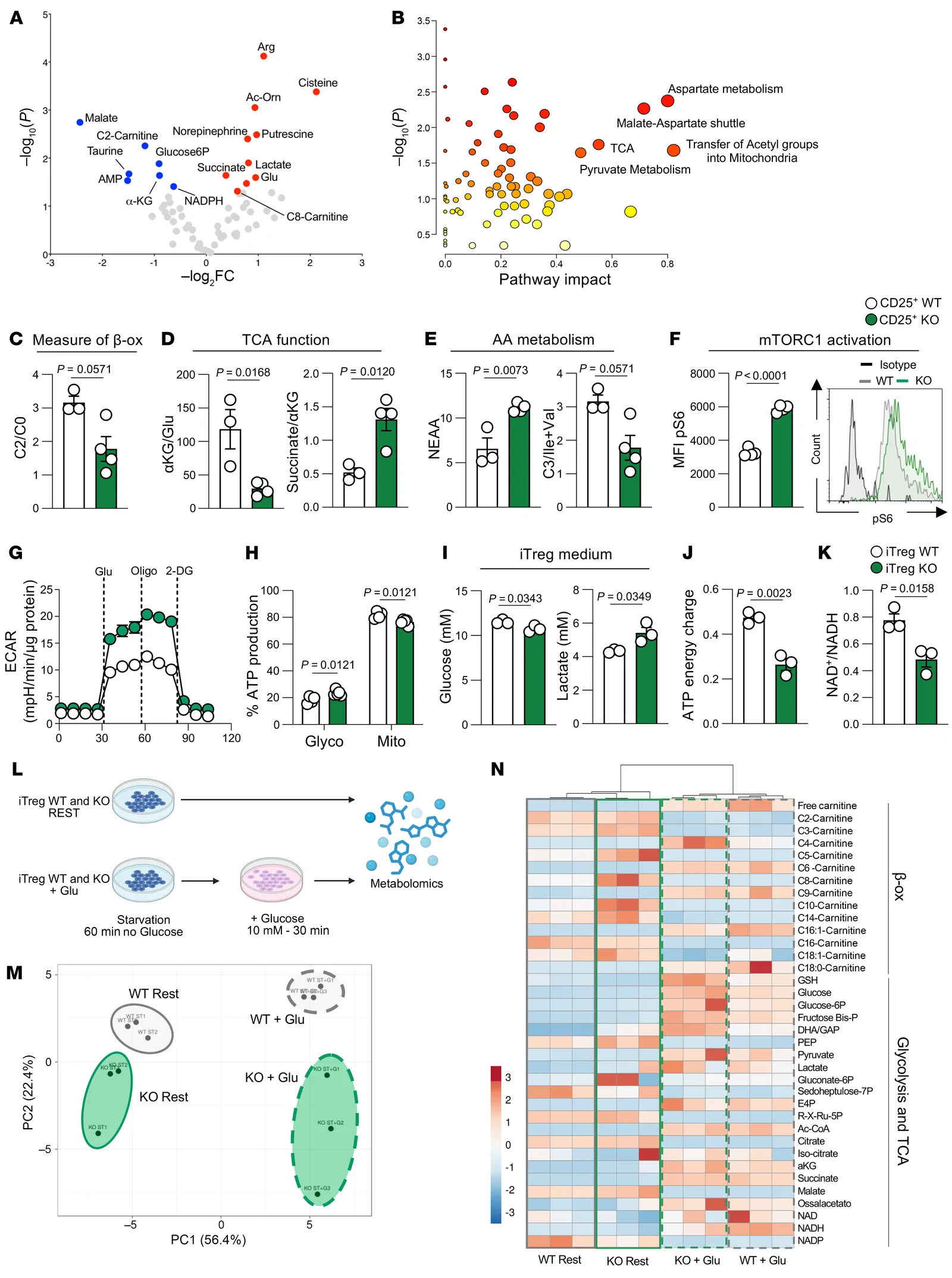
*Srebp1c* deficiency rewires Treg energetic metabolism toward glycolysis. (**A**) Volcano plot from targeted metabolomics of CD25^+^ cells; statistically significant down- and upregulated metabolites (log_2_FC ± 0.5 and *P* < 0.05) are depicted in blue and red, respectively. (**B**) Pathway analysis of significantly different metabolites performed by MetaboAnalyst from targeted metabolomics. Pathway impact (*x* axis) and significance (–log_10_*P* value (*y* axis) are represented. (**C**–**E**) Ratios between metabolites form targeted metabolomics: acylcarnitine (C2)/free carnitine (**C**), αketoglutarate/glutamate and succinate/αketoglutarate (**D**), and nonessential amino acids and acylcarnitine (C3)/branched amino acids isoleucine and valine (**E**). (**F**) Median fluorescence intensity (MFI) of the phosphorylated form of S6 tested by flow cytometry on CD25^+^ cells; representative histograms from flow cytometry analysis are shown. (**G** and **H**) ECAR analysis by glucose stress test (**G**) and ATP production by ATP rate assay from glycolysis and OXPHOS (**H**) tested by Seahorse on iTregs. (**I**) Glucose and lactate concentration (mM) by metabolomic analysis of supernatants of iTregs cultured for 4 days. (**J** and **K**) Ratios from targeted metabolomics of CD25^+^ cells: ATP energy charge calculated as ([ATP] + 0.5 × [ADP])/([AMP] + [ADP] + [ATP]) and NAD^+^/NADH. (**L**) iTregs were cultured in a glucose-free medium for 60 minutes, after which they were processed either immediately or after a 30-minute culture in a glucose-containing medium (10 mM) for metabolomic analysis. (**M**) PCA analysis from iTregs in glucose-free medium (rest, gray and green full line) or after the addition of glucose (10 mM final concentration; gray and green dotted line). (**N**) Heatmap of target metabolomics of iTregs in glucose-free medium or after the addition of glucose. Metabolites are clustered in rows according to metabolic pathways of β-oxidation, glycolysis, and TCA. Normalized values were used for the analysis by the ClustVis web tool (https://biit.cs.ut.ee/clustvis/). *N* = 3–6/ group. Data are presented as mean ± SEM. Statistical analysis has been performed with unpaired nonparametric *t* test (**C**–**F**, and **I**–**K**) and 2-way ANOVA (**H**).

Because Treg function is intrinsically linked to cellular energetic asset, we next investigated whether *Srebp1c* deficiency affected Treg functionality by perturbing intracellular metabolism. PCA analysis from targeted metabolomics showed a clear different metabolic signature in CD4^+^CD25^+^ Tregs purified from the spleens of *Srebp1c*-KO mice compared with those of WT mice (Supplemental Figure 5A). Among the most representative energetic metabolites, *Srebp1c*-KO Tregs exhibited an increased level of lactate, coupled to reduced levels of glucose-6 phosphate, α-ketoglutarate, malate, and C2-carnitine, as compared with WT Tregs (Figure 3A). Indeed, pyruvate metabolism, TCA, and transfer of acetyl groups into mitochondria were the most affected pathways in *Srebp1c*-deficient Tregs compared with those in WT cells (Figure 3B), suggesting a remodeling of energy metabolism. In parallel, metabolite set enrichment analysis revealed that, beyond pathways related to energetic metabolism (e.g., anaerobic glycolysis, pyruvate metabolism, TCA, and transfer of acetyl groups into mitochondria), those related to amino acids (phenylalanine and tyrosine metabolism, valine, leucine and isoleucine degradation), nucleotides (purine metabolism) and lipids (arachidonic acid and sphingolipid metabolism) were affected by *Srebp1c* deficiency (Supplemental Figure 5B), indicating a broad metabolic rewiring in *Srebp1c*-deficient Tregs. This was accompanied by a significant reduction in the α-ketoglutarate/glutamate ratio and an increase in the succinate/α-ketoglutarate ratio (Figure 3D), suggesting alterations in TCA cycle metabolism. In turn, the increase in nonessential amino acids, together with a trend toward reduced ratio between C3-acylcarnitines and the branched-chain amino acids isoleucine and valine (Figure 3E), was consistent with alterations in amino acid metabolism associated with the metabolic rewiring observed in *Srebp1c*-KO Tregs. Overall, these metabolic changes suggest a potential shift in cellular energy metabolism, possibly reflecting a rebalancing between glycolytic and mitochondrial pathways. Finally, we observed increased pS6 phosphorylation, a downstream target of mTORC1, in *Srebp1c*-KO cells compared with WT CD4^+^CD25^+^ cells (Figure 3F), suggesting a dysregulated immunometabolic phenotype.

In line with these results, glycolysis stress test by Seahorse metabolic assays showed an increased extracellular acidification rate (ECAR) in *Srebp1c-*KO iTregs after the administration of glucose and oligomycin (Figure 3G), indicating increased glycolysis and glycolytic capacity (Supplemental Figure 5, C and D) compared with that of WT controls. Accordingly, glycolytic ATP production was higher in *Srebp1c-*KO compared with WT iTregs, while ATP from OXPHOS was reduced in KO compared with WT iTregs (Figure 3H), corroborating that *Srebp1c* deficiency reprograms Tregs toward a more glycolytic phenotype. These results were confirmed by the detection of reduced glucose and increased lactate concentration in the culture medium of *Srebp1c*-KO compared with WT iTregs (Figure 3I). In agreement with this finding, a reduced ATP energy charge (Figure 3J), paralleled by the reduction of the NAD+/NADH ratio (Figure 3K) by targeted metabolomic analysis of iTregs, corroborated the increased glucose flux through the glycolytic pathway. We excluded that these changes were driven by a proliferative advantage in *Srebp1c*-KO Tregs, as similar expression of Ki67 and rates of in vitro proliferation were detected in both ex vivo Tregs (Supplemental Figure 4, C–E) and iTregs (Supplemental Figure 4, F–H).

To better characterize the glucose dependence of *Srebp1c*-KO iTregs, targeted metabolomic analysis of iTregs was performed in a glucose-free medium (1 h, rest) and after incubation with glucose re-supplementation (10 mM, + Glu) for 30 minutes (Figure 3L). *Srebp1c* deficiency was associated with a distinct reprogramming of the metabolome relative to WT iTregs, a change that became more pronounced upon glucose exposure (Figure 3M). Notably, *Srebp1c*-KO iTregs accumulated higher levels of medium-chain acyl-carnitines (C5, C8, C10) compared with WT cells under glucose-free condition (KO rest vs. WT rest) (Figure 3N), suggesting alterations in FA β-oxidation metabolism. In contrast, upon glucose supplementation, an increase in glycolytic flux was highlighted by the higher abundance of glycolytic intermediates (glucose-6P, fructose-1,6 biphosphate, DHA/GAP) in *Srebp1c*-KO compared with WT iTregs (Figure 3N), consistent with enhanced glycolytic activity. This energetic shift was specific to Tregs, as conventional CD4^+^CD25^–^ T cells from *Srebp1c-*KO mice exhibited a metabolic profile superimposable on that of WT cells (Supplemental Figure 5, E and F) and similar glycolytic metabolism (Supplemental Figure 5G). In line with a similar metabolic profile, in vitro proliferation of *Srebp1c*-KO CD4^+^ T cells was not different from that of WT cells (Supplemental Figure 5H).

Taken together, these results indicate that *Srebp1c* deficiency causes a specific energetic reprogramming of Tregs, shifting cellular metabolism toward a more glycolytic phenotype.

### Srebp1c preserves immunometabolism of Tregs by controlling cellular lipid homeostasis.

**Figure 4:**
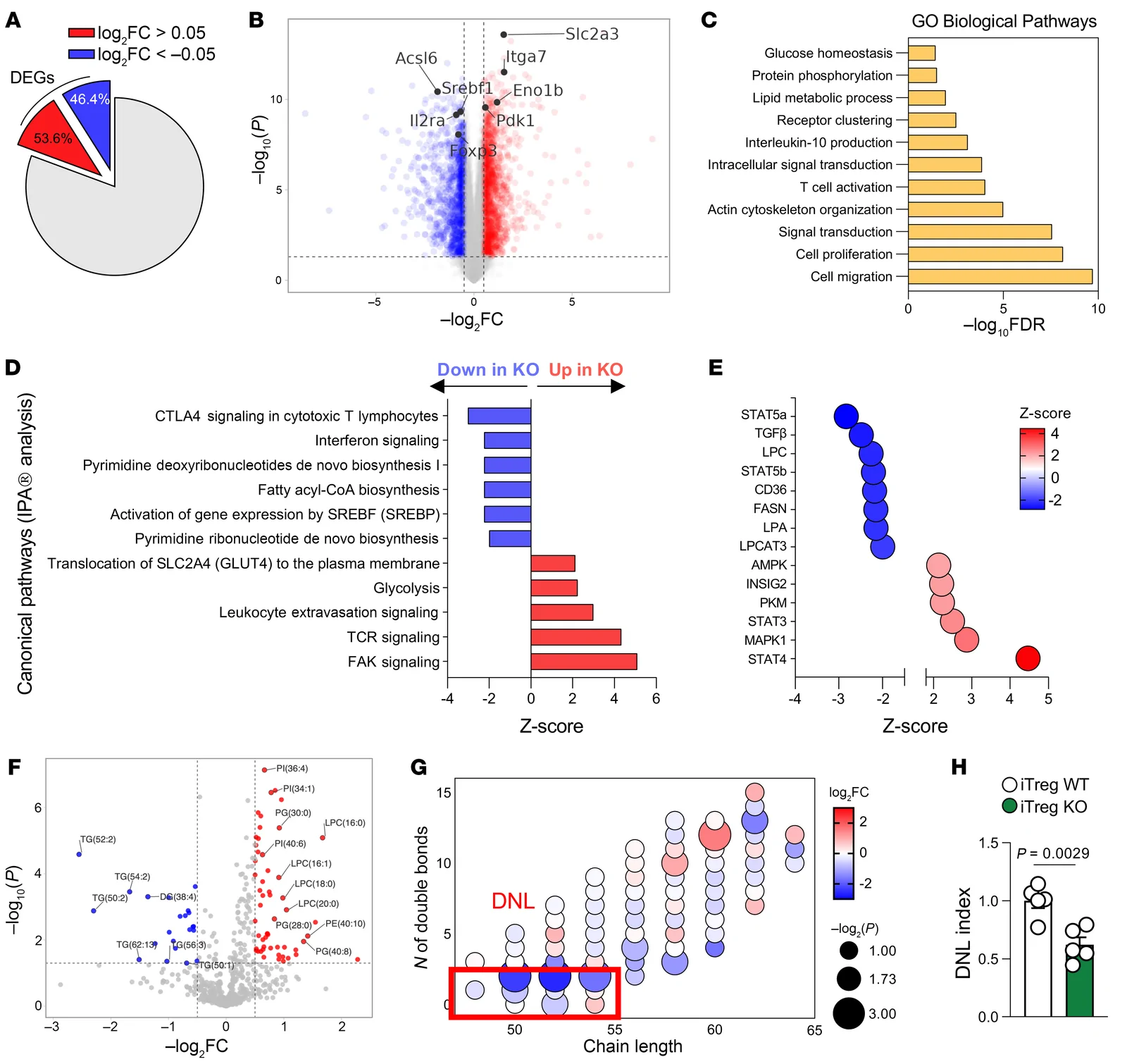
SREBP1c is an intrinsic determinant of Treg immunometabolic phenotype. (**A**) Pie chart of the distribution of significantly (colored) or not (gray) different expressed genes (DEGs) between upregulated (log_2_FC > 0.05, red) and downregulated (log_2_FC < 0.05, blue) genes. (**B**) Volcano plot of RNA-seq showing the most relevant DEGs (upregulated genes are in red, and downregulated genes are in blue). (**C**) Gene ontology analysis of biological process (GO BP) from David database analysis of DEGs from RNA-seq of *Srebp1c*-KO iTregs compared with WT Tregs. Enriched pathways in *Srebp1c*-KO iTregs are expressed as –log_10_FDR > 1.3. (**D**) *Z* score analysis using IPA software of canonical pathways from IPA analysis of significant DEGs. (**E**) Z score analysis using IPA software of upstream regulators analysis of IPA analysis of significant DEGs. (**F**) Volcano plot showing the quantified lipid species (sum notation) in iTregs. Blue and red represent the downregulated and upregulated lipid species in *Srebp1c*-KO iTregs, respectively [log_2_FC ± 0.5 and –log_10_(*P* value) > 1.3]. (**G**) Bubble plot of TG species compared by number of carbons (chain length) and number of double bonds (*N* double bonds). Bubble dimension is a function of –log_10_ of *P* value, while colors represent log_2_FC (increase in red, decrease in blue). In the red box are TGs with fewer than 54 carbons and 3 double bonds as indicators of de novo lipogenesis (DNL). (**H**) De novo lipogenesis index is calculated as the sum of TGs with fewer than 54 carbons and 3 double bonds in *Srebp1c*-KO and WT iTregs normalized to the WT mean. Lipidomics and RNA-seq were performed on 5 biological replicates/group. In **H**, data are presented as mean ± SEM, and statistical analysis has been performed with unpaired nonparametric *t* test.

To elucidate the molecular mechanisms involved, we performed RNA-seq from *Srebp1c* WT and -KO iTregs. PCA analysis revealed a different transcriptome profile in *Srebp1c*-KO Tregs compared with that in WT cells (Supplemental Figure 6A). We detected 2,615 differently expressed genes (DEGs), 46.4% of which were downregulated and 53.6% were upregulated genes with a log_2_fold change of 0.5 (Figure 4A). Among the top DEGs, we observed the downregulation of genes involved in lipid metabolism, including *Srebf1*, which encodes the SREBP1a and SREBP1c isoforms, and *Acsl6*, which encodes acyl-CoA synthetase long-chain family member 6. Conversely, several upregulated genes were associated with glycolysis, including *Slc2a3* (glucose transporter 3, that supports glucose uptake), *Eno1b* (enolase 1b, involved in glycolysis by phosphoenolpyruvate production), and *Pdk1* (pyruvate dehydrogenase kinase 1, that switches metabolism toward the Warburg effect) (Figure 4B). In parallel, genes strictly connected to Treg functionality, including *Il2r* (IL-2 receptor, CD25) and *FoxP3* were downregulated in *Srebp1c*-KO Tregs, while *Itga7* (integrin subunit α 7, required for cell migration) and *Il7r* (IL 7 receptor, required for T cell development and proliferation) were upregulated (Figure 4B). Overall, these findings confirmed — at transcriptional level — a shift from lipid to glycolytic metabolism and to impaired Treg phenotype. Gene Ontology of Biological process analysis of DEGs identified pathways belonging to glucose homeostasis and lipid metabolism as well as immune function, such as IL-10 production, T cell activation, signal transduction, and migration, among the most affected by *Srebp1c* deficiency (Figure 4C). In more detail, genes related to lipid metabolism pathways (FA transport, lipid biosynthetic process, PL homeostasis and FA metabolic process) were mostly downregulated (Supplemental Figure 6B), while those related to energetic metabolism (glycolysis, response to insulin and carbohydrate metabolic process) (Supplemental Figure 6B) were upregulated in *Srebp1c*-KO compared with WT Tregs. In line with the findings presented above, genes involved in cell migration and T cell activation were mostly upregulated (Supplemental Figure 5B), while those related to Treg differentiation and IL-10 production were mostly downregulated (Supplemental Figure 6C). Unsupervised canonical pathway analysis by Ingenuity Pathway Analysis (IPA) showed a predicted activation (*z* score >2) of pathways associated with translocation of SLC2A4 to plasma membrane, glycolysis, leukocyte extravasation, and focal adhesion kinase signaling (a tyrosin kinase that mediates intracellular signal transduction and regulates cell migration) (Figure 4D), with the latter in line with previously reported data on migration of *Srebp1c-*KO Tregs (Figure 2, L and M, and Supplemental Figure 4, A and B). In contrast, CTLA-4 and interferon signaling, together with fatty acyl-CoA biosynthesis and activation of SREBF gene pathways (Figure 4D), were predicted to be downregulated (*z* score < –2), further supporting that *Srebp1c*-dependent circuits are normally activated in Tregs. Other pathways that emerged to be downregulated by IPA canonical pathway analysis were the ribo- and deoxynucleotide synthesis. Given that the pentose phosphate pathway (PPP) is essential for nucleotide synthesis — providing ribose-5-phosphate for purine and pyrimidine production — and that its rate-limiting glucose-6-phosphate dehydrogenase is regulated by SREBP1c, we investigated whether PPP impairment contributed to the metabolic phenotype of *Srebp1c*-deficient Tregs. For this purpose, we traced D-glucose-1,2-^13^C_2_ flux to specifically track the PPP (Supplemental Figure 6D). *Srebp1c*-KO iTregs exhibited increased incorporation of labeled glucose-derived carbons (Supplemental Figure 6E) and accumulation of glycolytic intermediates, such as dihydroxyacetone phosphate/glyceraldehyde 3 phosphate (DHAP/GAP) and phosphoenolpyruvate (Supplemental Figure 6E) compared with WT iTregs, confirming increased glucose uptake and glycolytic pathway under *Srebp1c* deficiency. In addition, we detected increased labeled carbons in the metabolites of the nonoxidative branch of the PPP, such as sedoheptulose 7 phosphate (S7P) and erythrose 4-phosphate (E4P) (Supplemental Figure 6E), while no differences were detected in the metabolites of the oxidative phase (gluconate-6 phosphate and ribulose-5 phosphate, data not shown). These findings indicate that the increased nonoxidative branch of the PPP likely resulted from the GAP shunt due to heightened glycolytic flux, rather than direct impairment of the PPP. Additionally, elevated ribo- and deoxynucleotides in Srebp1c-KO iTregs suggest more purine and pyrimidine precursors, possibly suggesting an impaired de novo nucleotide synthesis.

In line with previous findings, analysis of upstream regulators by IPA (Figure 4E) identified Treg-suppressive function signaling pathways (*z* score < 2 for STAT5a/b and TGF-β) and lipid metabolism (*z* score < 2 for FASN and > 2 for INSIG2, involved in the retention of SREBP isoforms in the endoplasmatic reticulum) inhibited, while, in contrast, STAT3/4 and MAPK1 pathways were predicted to be activated with a *z* score > 2, together with glycolysis (*z* score > 2 for PKM, pyruvate kinase involved in glycolysis) and catabolic pathway (*z* score > 2 for AMPK activation) (Figure 4E). Instead, CD36 was predicted to be inhibited (*z* score < 2), and this was confirmed with analysis of mRNA and protein expression (Supplemental Figure 6, H and I), suggesting an impaired compensation from FA uptake. Indeed, when glycolytic activity was tested in the presence of palmitate, with or without etomoxir, an inhibitor of CTP1α (at a concentration of 5 μM that does not affect Treg polarization and viability, ref. 15; Supplemental Figure 6J), supplementation with palmitate reduced glycolysis in WT cells, an effect driven by increased FAO, as suggested by the reversion of the energetic phenotype in the presence of etomoxir (Supplemental Figure 6K). However, in *Srebp1c*-KO iTregs, palmitate supplementation, with or without etomoxir, was similar to that of control cells (*Srebp1c*-KO iTregs in the presence of BSA), functionally limiting compensation for extracellular FA uptake under *Srebp1c*-deficient conditions (Supplemental Figure 6K).

To clarify how Srebp1c deficiency affected Treg lipid metabolism, we conducted lipidomics analysis on *Srebp1c*-WT and KO iTregs. 1,255 lipid species were detected in WT and *Srebp1c*-KO iTregs (Supplemental Figure 7A), with the most abundant belonging to the family of phosphatidylethanolamines (PE; 24.9%), PC (20.6%), TG (16.4%), and phosphatidylinositols (PI; 11.4%) (Supplemental Figure 7B). *Srebp1c*-KO iTregs showed a profound difference in lipidome composition compared with WT cells (Supplemental Figure 7C), and, despite no difference in the total lipid abundance (Supplemental Figure 7D), 21.75% of lipid species were differently expressed (265 lipids), with 16.50% being upregulated and 5.25% downregulated (Supplemental Figure 7E). Within the lipid species, PLs, such as LPC and PI species, were increased, while TGs were reduced (Figure 4F) in *Srebp1c*-KO iTregs compared with WT cells. Focusing on downregulated lipid species, we noticed that several TGs consisted mostly of chains with fewer than 54 carbons and 3 saturations, indicating FA chains derived from de novo synthesis (Figure 4G). Therefore, we compared the sum of these TGs, as an indicator of de novo lipid synthesis (DNL index); it showed a significant reduction in FA synthesis in *Srebp1c*-KO iTregs compared with WT iTregs (Figure 4H), thus confirming the crucial role of SREBP1c as a modulator of FA metabolism in Tregs. By combining transcriptomics and lipidomics, we concluded that *Srebp1c* deficiency profoundly affects Treg immunometabolism, impairing suppressive function and shifting the energetic machinery toward glycolysis as a possible compensatory response to impaired FA synthesis.

### Srebp1c deficiency remodels Treg PL composition and metabolism.

**Figure 5:**
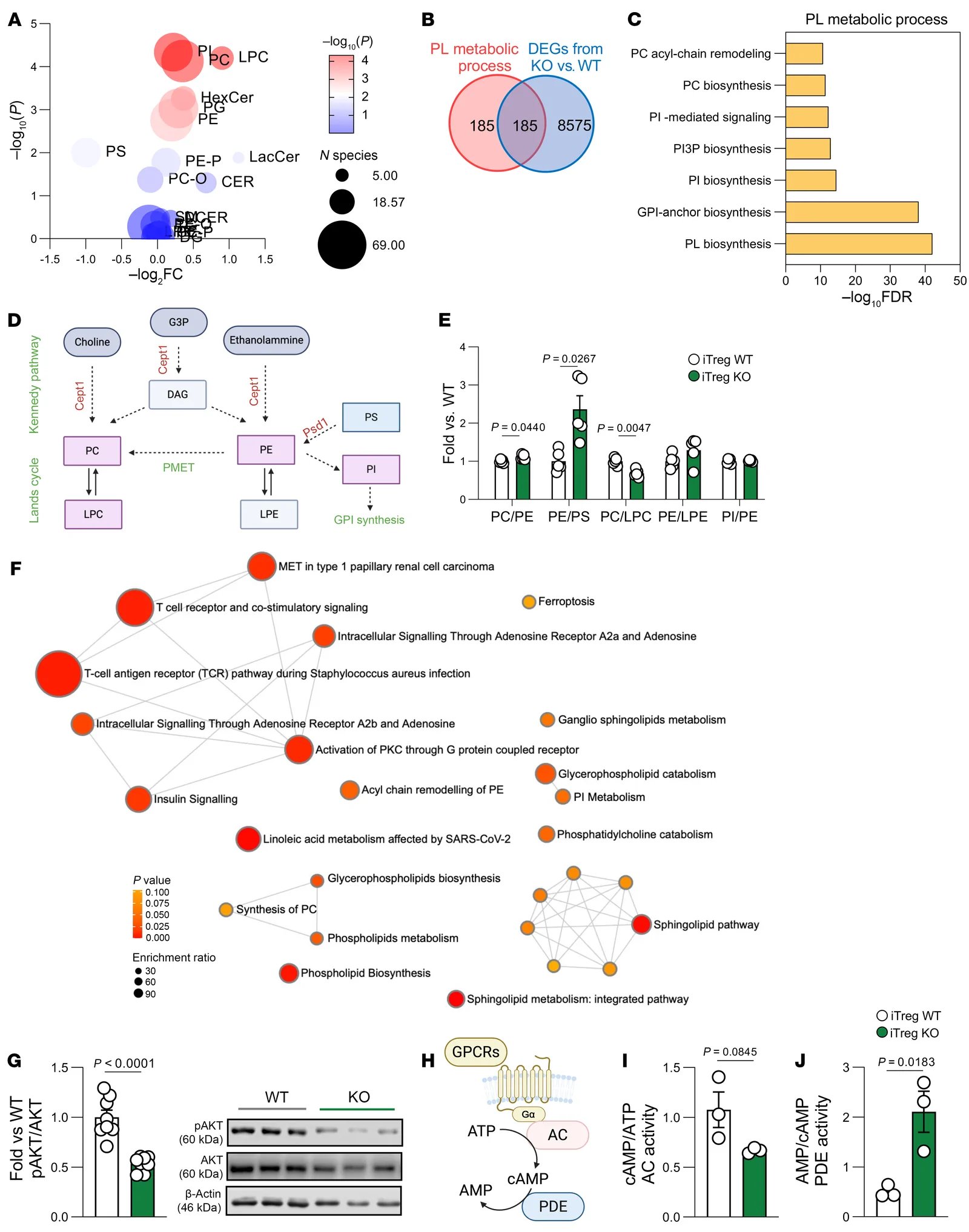
*Srebp1c* deficiency affects phospholipid metabolism. (**A**) Bubble plot of lipid classes presented as log_2_FC of their abundance (ng/mmol DNA of lipid species sum) versus the –log_10_ of *P* value. Color represents the –log_10_ of *P* value, while the size represents the number of lipid species for each class. (**B**) The Venn diagram represents the shared genes between our RNA-seq dataset on Tregs and the list of genes listed under the term “phospholipid metabolic process” (GO term 0006644). (**C**) GO BP analysis of **B**. Enriched pathways are expressed as –log_10_FDR > 1.3. (**D**) Representation of phospholipid metabolism; squares represent lipid classes from lipidomics (light blue indicates reductions in KO, while light red increases; gray indicates no difference). (**E**) Fold difference in phospholipid classes from lipidomic analysis. The ratio between the sum of lipid species for each class is presented and normalized to the mean of the WT values. (**F**) Enrichment analysis from a RaMP-DB pathway–based (https://www.metaboanalyst.ca/) library by MetaboAnalyst of pathways affected by significantly different lipid species. (**G**) Quantification of AKT activation as pAKT versus AKT ratio from Western blot analysis. Data are corrected on β-actin abundance; a representative Western blot for pAKT, AKT, and β-actin is shown. (**H**) Graphic representation of GPCR intracellular activation leading to cAMP generation from ATP through the activation of adenylate cyclase (AC). cAMP can be degraded to AMP by phosphodiesterase (PDE) enzymes. (**I**) Ratio between cAMP/ATP normalized to WT from metabolomic analysis of iTregs (AC activation). (**J**) Ratio between AMP/cAMP normalized to WT from metabolomic analysis of iTregs (PDE activation). Data in **A**, **E**, and **F** derive from lipidomic analysis (5 biological replicates/group); data in **B** and **C** are from RNA-seq analysis (5 biological replicates/group); **G**, *n* = 8 samples/group; data in **I** and **J** derive from metabolomics of iTregs (3 biological replicates/group). Data are presented as mean ± SEM. Statistical analysis has been performed using 2-way ANOVA (**E**) and unpaired nonparametric *t* tests (**G**–**J**).

In parallel with a reduction of TGs, we also observed an accumulation of PL species in *Srebp1c-*KO compared with WT iTregs (Figure 4F). This evidence aligns with previous observations showing that PLs are enriched in human Tregs and are associated with their suppressive phenotype (16). To better explore this difference, we investigated lipid distribution at the level of lipid classes, reporting a significant increase of several PL classes, such as glycophospholipids (LPC, PC, and PI) and ceramides (CER and LacCer), with a reduction in phosphatidylserines (PS) in *Srebp1c-*KO compared with WT iTregs (Figure 5A). Furthermore, we observed a general reduction of the 16:1/16:0 and C18:1/C18:0 desaturation index, particularly evident in LPC, PG, PI, and PS as well as LPC, PC, PE, and SM lipid classes, respectively (Supplemental Figure 7, F and G), indicating a reduced activity of desaturase enzymes that are controlled by SREBP1c and have been shown to contribute to Treg phenotype (17). Given that most of the observed differences were among upregulated PLs, we delved into the mechanisms that control their metabolism in Tregs. To this end, we screened RNA-seq data from iTregs for genes associated with the *phospholipid metabolic process* term (GO:0006644), identifying 185 DEGs shared across both datasets (Figure 5B), belonging to pathways related to glycosylphosphatidylinositol (GPI) anchors, PI and PC biosynthetic process, and PC acyl-chain remodeling (Figure 5C), supporting a contribution of SREBP1c to PL metabolism in Tregs.

PL homeostasis is a complex and coordinated process, controlled at different levels of PL de novo synthesis, conversion, and remodeling (18) (Figure 5D). To understand whether SREBP1c modulates these processes, we analyzed PL relative abundance and observed a significant increase in PC/PE and PE/PS ratios in *Srebp1c*-KO compared with WT iTregs (Figure 5E), indicating an enhanced conversion of PL to PC. In contrast, the ratio between PC and their respective Lyso-PC forms (LPC), but not PE/LPE, was significantly reduced in *Srebp1c*-KO compared with WT cells (Figure 5E), suggesting that *Srebp1c* deficiency might be associated with an impaired recycling of PC from LPC, resulting in an imbalanced PL homeostasis. To determine how this altered balance in PL composition would affect Treg biology, we performed enrichment analysis on significantly different lipid species. In parallel to enriched pathways related to PL metabolism (PL biosynthesis, catabolism, acyl chain remodeling; Figure 5F), this analysis showed the enrichment of specific pathways associated with Treg metabolism and function, such as insulin signaling, T cell receptor (TCR), and adenosine receptors signaling pathways, among the pathways most affected by *Srebp1c* deficiency (Figure 5F). To functionally prove this prediction, we investigated the activation of AKT, a downstream target of both insulin receptor and TCR and costimulatory signaling; pAKT/AKT was significantly reduced in *Srebp1c*-KO iTregs compared with WT iTregs (Figure 5G). In line with this, several intracellular pathways associated with TCR signaling, such as LCK, LAT, and PIK3R2, were predicted to be downregulated in KO Tregs by RNA-seq (Supplemental Figure 8A). More importantly, a key Treg immunosuppressive mechanism, the adenosine pathway, was predicted to be affected by lipid remodeling induced by *Srebp1c* deficiency through the G protein–coupled receptors (GPCRs) ADORA2a and ADORA2b intracellular signaling (Figure 5F). To explore this, we measured the ratio between cAMP and ATP, an indicator of adenylate cyclase (AC) activity, that is activated downstream to ADORA2a and ADORA2b activation (Figure 5H). Our findings showed that the cAMP/ATP ratio was reduced in *Srebp1c*-KO iTregs (Figure 5I), suggesting reduced adenosine signaling through these receptors compared with WT cells. Notably, this reduction was associated with increased phosphodiesterase (PDE) activity, measured as the AMP/cAMP ratio, which was elevated in *Srebp1c*-KO compared with WT iTregs (Figure 5J). To corroborate these findings, we investigated genes associated with adenosine metabolism (Supplemental Figure 8B) in the RNA-seq dataset. We reported that genes involved in adenosine synthesis (*Nte, Entpd1, Dpp4*) and transport (*Slc29a3, Slc28a2*) were upregulated, whereas genes mediating adenosine catabolism (*Pnp, Ampd, Ada*) were downregulated, with the exception of *Akd*, which was upregulated (Supplemental Figure 8C). In line with the predicted reduced activity of AC and increased PDE activity (Figure 5, I and J), the expression of *Adcy9*, an isoform of AC involved in immunosuppressive cAMP production (19), was reduced, while that of *Pde3b*, an enzyme that degrades cAMP to AMP and associated with Treg-suppressive function (20, 21), was upregulated (Supplemental Figure 8C).

Collectively, these results suggest that SREBP1c preserves PL homeostasis in Tregs and that remodeling of PL metabolism and composition impairs the metabolism of the immunosuppressive adenosine pathway.

### SREBP1c-dependent modulation of membrane PL affects extracellular adenosine metabolism.

**Figure 6:**
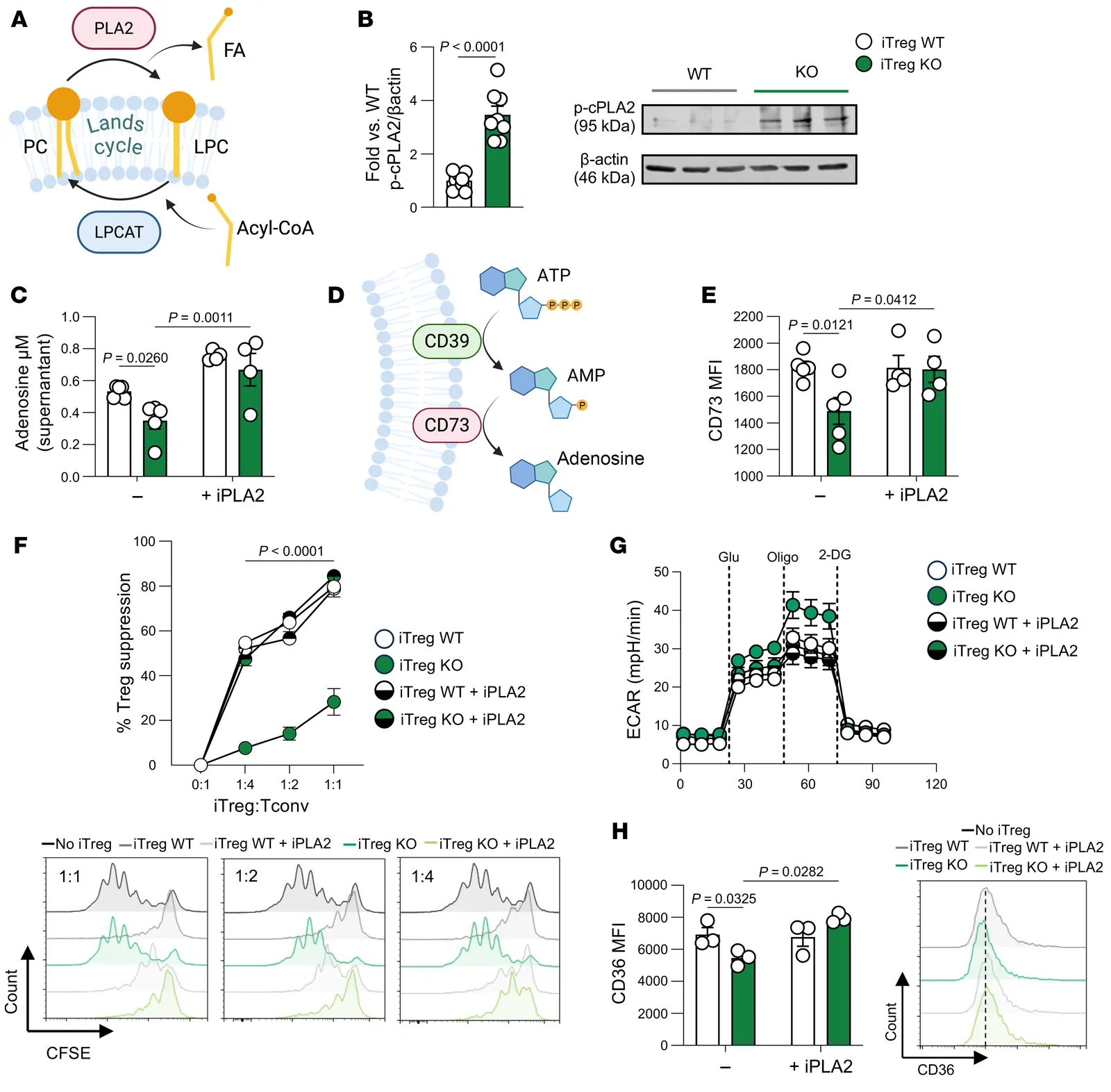
*Srebp1c*-mediated phospholipid homeostasis supports adenosine metabolism. (**A**) Graphic representation of acyl-chain remodeling of phospholipids by the Lands cycle: phospholipase A2 (PLA2) removes an acyl-CoA from a phosphatidylcholine (PC), generating a lysophosphatidylcholine (LPC); then, the LPC acyltransferase (LPCAT) transfers an Acyl-Coa to an LPC to form a PC. (**B**) Quantification of p-cPLA2α by Western Blot analysis. Data are corrected on β-actin abundance; a representative Western blot for p-cPLA2α and β-actin is shown. (**C**) Concentration of adenosine in the supernatants of iTregs treated or not with the cPLA2α inhibitor pyrrophenone (10 nM for 3 h). (**D**) Graphic representation of adenosine metabolism: extracellular ATP is hydrolyzed by CD39 to AMP (via an ADP intermediate), which is then converted to adenosine by CD73. (**E**) Expression of CD73 by flow cytometry in iTregs treated or not with the cPLA2α inhibitor pyrrophenone (10 nM for 3 h). (**F**) Suppression of Tconv proliferation by iTregs pretreated or not with the cPLAα inhibitor MAFP (5 μM for 3 h) at increasing ratios with T conventional cells (0:1 indicates that no Tregs were used, while 1:4, 1:2, and 1:1 indicate the proportion of iTregs to Tconv) for 3 days. Representative histograms from flow cytometry are shown. (**G**) ECAR analysis by glucose stress test tested by Seahorse on iTregs pretreated or not with the cPLA2α inhibitor pyrrophenone (10 nM for 3 h). (**H**) Expression of CD36 in iTreg pretreated or not with the cPLA2α inhibitor pyrrophenone (10 nM for 3 h). Representative histograms from flow cytometry analysis are shown. *N* = 3–8/group. Data are presented as mean ± SEM. Statistical analysis has been performed with unpaired nonparametric *t* test (**B**) or 2-way ANOVA (**C**, **E**, **F**, and **H**).

Considering the effect of *Srebp1c* deficiency on the PC-to-LPC balance, we focused on the mechanism of PC remodeling via the Lands cycle. In this process, LPC acyltransferase (LPCAT) enzymes add an acyl-CoA to LPC, and the phospholipases A2 (PLA2) in turn release an acyl chain from PC to generate a LPC (22–25) (Figure 6A). Compared with WT iTregs, *Srebp1c*-KO iTregs presented an increased expression of the cPLA2α, the dominant member of the PLA2 family, with a 95% homology with the human protein and activated after cell stimulation (26) (Figure 6B), suggesting an increased release of an acyl-CoA from a PC to generate an LPC, in line with results from lipidomics (Figure 5A). No difference was instead reported in the expression of LPCAT3 (Supplemental Figure 9A), which controls the reacylation step in the Lands cycle (Figure 6A). Therefore, to establish a link between increased cPLA2α activity and impaired adenosine metabolism (Supplemental Figure 8, B and C), we measured the levels of extracellular adenosine in the culture supernatant of iTregs. This was performed in the presence or not of a specific inhibitor of cPLA2α activity (27). *Srebp1c*-KO iTregs showed a reduced extracellular concentration of adenosine compared with WT cells, and cPLA2α inhibition increased adenosine levels in the supernatant of both WT and *Srebp1c*-KO iTregs (Figure 6C). As extracellular adenosine mainly derives from the consecutive conversion of ATP to AMP and to adenosine by the enzymatic activity of the ectonucleotidases CD39 and CD73, respectively (Figure 6D), we evaluated their abundance on iTregs. Expression of CD73, but not CD39, was significantly reduced in *Srebp1c*-KO compared with WT iTregs (Supplemental Figure 9B). Notably, a similar pattern was observed in vivo, with a significant reduction in CD73 — but not CD39 — expression on CD4^+^CD25^hi^FOXP3^+^ Tregs from *Srebp1c*-deficient mice compared with WT mice (Figure 2J and Supplemental Figure 9, C and D), whereas conventional CD4^+^CD25^–^ T cells showed no changes (Supplemental Figure 9, E and F). Pharmacological inhibition of cPLA2α restored CD73 surface expression in *Srebp1c*-KO iTregs to levels comparable to those of WT cells (Figure 6E), providing a mechanistic basis for the increased adenosine levels observed under these conditions. More importantly, cPLA2α inhibition rescued the suppressive function of *Srebp1c-*deficient iTregs to similar levels of WT cells, when iTregs treated or not with cPLA2α inhibitor were cocultured with Tconv (Figure 6F). This result linked the reduced CD73-adenosine metabolism to the decreased suppressive function of *Srebp1c*-KO iTregs driven by impaired PL remodeling. Finally, cPLA2α inhibition also reduced the glycolytic flux of *Srebp1c*-KO iTregs to levels comparable to those of WT cells (Figure 6G), possibly as a consequence of restored CD36 expression on the plasma membrane after cPLA2α inhibition, suggesting that PL remodeling can also modulate the energetic phenotype of Tregs.

Together, these results indicated that altered PL metabolism affects the energetic and suppressive phenotypes of Tregs by modulating the adenosine pathway, a mechanism controlled by SREBP1c.

## Discussion

In this study, we explored the role of SREBP1c, the key regulator of cellular FA metabolism, in Treg biology. We found that this TF, beyond classical metabolic checkpoints, influences PL composition, which is essential for Tregs’ immunosuppressive and energy-related functions. We showed that *Srebp1c* deficiency impairs Treg suppression and affects their frequency and phenotype in vivo. Although SREBP1c is highly expressed in the liver, and *Srebp1c*-KO mice have reduced plasma lipid levels compared with WT (11), we ruled out that the Treg phenotype was driven by alterations in systemic metabolism. Indeed, when *Srebp1c*-KO Tregs were injected into normolipidemic mice, amelioration of EAE severity was partially lost compared with mice receiving WT Tregs, confirming a reduced suppressive activity of *Srebp1c*-deficient Tregs independently of plasma lipid levels. Consistent with this, Tregs generated in vitro from conventional CD4^+^ T cells (iTregs) also exhibited an impaired suppressive function, further excluding a major effect of systemic metabolism and yet supporting an intrinsic role for intracellular SREBP1c in Treg biology. Indeed, *Srebp1c* deficiency profoundly rewires energy metabolism toward glycolysis in Tregs, consistent with the notion that Tregs primarily rely on lipid oxidation rather than glycolysis to support their immunosuppressive activity (28). In line with this, the enhanced migration of *Srebp1c*-KO Tregs confirms our previous data that glycolysis supports Treg migration (2). Unexpectedly, palmitate supplementation failed to reverse the glycolytic flux of *Srebp1c*-KO Tregs in line with reduced CD36 expression, suggesting that in our experimental setting, reduced FA synthesis was not compensated by increased FA uptake, as previously shown (29, 30). This metabolic phenotype is consistent with impaired Treg suppressive function and aligns with observations that CD36 deficiency selectively reduces the abundance and suppressive activity of intratumoral Tregs (31). While confirming prior evidence of the important role of FA metabolism, our data added a layer of complexity to the immunometabolic reprogramming of Tregs, as *Srebp1c* deficiency resulted in a distinct lipidomic signature characterized by PL remodeling and alterations in multiple glycerophospholipid pathways. Indeed, PL enrichment has already been reported in human Tregs compared with Tconv, with the presence of long, unsaturated FA chains that affect plasma membrane fluidity (16). We ascribed the phenotype of *Srebp1c*-KO Tregs to an impaired remodeling of PC acyl chains due to increased expression of cPLA_2_α and LPC accumulation. In contrast, although RNA-seq analysis predicted that LPCAT3 expression would be inhibited, possibly as a consequence of *Srebp1c* deficiency, since LPCAT3 controls upstream processing of SREBP1c (32), protein levels were unchanged, excluding a direct effect of *Srebp1c* deficiency on the acylation step of the Lands cycle (33).

Instead, our observation mirrors the phenotype reported in senescent CD8^+^ T cells within the tumor microenvironment, where STAT1/3 and MAPK1 signaling pathways cooperate to promote cPLA_2_α overexpression, leading to impaired lipid metabolism, energetic rewiring toward glycolysis, and reduced antitumor immunity (34). In line with this, *Srebp1c*-KO Tregs showed activation of the STAT3 and MAPK1 pathways, along with increased cPLA_2_α expression. Altogether, we speculate that cPLA2α overexpression may reflect a compensatory mechanism in response to reduced FA availability, whereby FAs are mobilized from PC to mitigate reduced synthesis and extracellular uptake. In turn, LPC accumulation reorganizes plasma membrane GPCRs, such as the adenosine receptors (35), affecting Th17 cell/Treg balance (36). In agreement with this, the altered lipidome of *Srebp1c-*KO Tregs also impaired intracellular signaling (as suggested by reduced pAKT activation) and, more specifically, the adenosine pathway, a key Treg suppressive mechanism (37). Adenosine activates ADORA2a and ADORA2b and the downstream cAMP signaling pathway in a paracrine and autocrine fashion, resulting in opposite effects: while cAMP activation has a general suppressive role, it enhances the proliferation and activity in Tregs (33, 37, 38, 39). In line with this, reduced cAMP generation in *Srebp1c*-KO Tregs aligns with their impaired suppressive function, as Treg-derived cAMP is a potent inhibitor of proliferation and IL2 synthesis in T cells (21), whereas FOXp3 controls repression of *Pde3B*, whose expression is increased in *Srebp1c*-KO Tregs. In addition to these intracellular mechanisms, we observed decreased extracellular adenosine in *Srebp1c*-KO Tregs, which we linked to reduced CD73 expression — the ecto-5′-nucleotidase that converts AMP to adenosine (40). This finding helps explain both the impaired intracellular phenotype of Tregs and their decreased suppressive ability. Interestingly, CD73 is anchored to the membrane by a GPI anchor, a single transmembrane domain that can link various proteins to the outer leaflet of the plasma membrane via their C-terminal domains (41). GPI shares a common backbone made up of an N-glycan structure and PLs (PE and PI); therefore, we argue that changes in PL metabolism in *Srebp1c*-KO Tregs compared with WT Tregs would affect specifically the expression of the GPI-anchor CD73.

Finally, we established a link between the impaired adenosine pathway and defective PL remodeling in *Srebp1c*-deficient Tregs, as inhibition of cPLA_2_α rescued extracellular adenosine levels and improved the suppressive and metabolic phenotypes of *Srebp1c*-KO Tregs.

In conclusion, we have identified Srebp1c as a key immunometabolic checkpoint of Treg function. By controlling FA metabolism, SREBP1c preserves the tolerogenic Treg phenotype and function at multiple levels, including maintenance of energetic and cAMP metabolism homeostasis (2–6, 20, 21, 28), corroborating several lines of evidence already reported in the literature. Our data extend the role of lipid metabolism in Tregs, showing that *Srebp1c* deficiency shifts metabolism toward glycolysis and to increased PL remodeling, as a possible attempt to compensate for reduced FA availability, ultimately impairing adenosine production through CD73 expression.

## Methods

### Mice.

B6;129S6-Srebf1^tm1Mbr^/J (strain 004365, RRID:IMSR_JAX:004365) and FoxP3-reporter B6.Cg-Foxp3^tm2Tch^/J (strain 006772, RRID:IMSR_JAX:006772) mice were purchased from The Jackson Laboratory. Up to 5 mice (according to body weight) were housed per conventional or individually ventilated cage and kept in a temperature-controlled environment (20°C ± 2°C, 50% ± 5% relative humidity) with a 12-hour light/dark cycle and free access to food and water.

For blood, lymph nodes and spleen collection, fed mice were euthanized by an overdose of CO_2_.

### Sex as a biological variable.

Results from females (*Srebp1c*-KO mice and littermate controls, and FoxP3-reporter mice) aged between 10 and 20 weeks are presented according to the increased severity of EAE challenge compared with males. Phenotypic and functional characterization was performed in males with similar findings.

### Tissue processing and Treg purification.

Lymphocytes were isolated from the thymus, lymph nodes, and spleen, and a uniform cell suspension was prepared by mashing the lymph nodes and/or spleens through a 70 μm cell strainer with 1 mL syringe plunger with PBS/2% FBS/2 μM EDTA PBS (MACS buffer). Cells were spun at 500*g* for 5 minutes, lysed with red blood cell lysis buffer for 5 minutes at 4°C (when depletion of red blood cells was required), suspended in MACS buffer, and counted. For Treg purification, the EasySep Kit (STEMCELL Technologies) was used according to the manufacturer’s instructions, resulting in separation of the CD4^+^CD25^+^ fraction and CD4^+^CD25^–^.

### Flow cytometry.

Immunophenotyping was performed on blood (50 μL) or cell suspensions (from thymus, spleen, lymph nodes, and cultured cells). Blood was incubated at room temperature for 30 minutes with the specific antibody mixture, and thereafter, samples were lysed and fixed according to the manufacturer’s instructions (1-step fix/lyse, eBioscience). Single-cell suspensions were incubated with antibody mixtures at 4°C for 30 minutes and then washed with MACS buffer. For intracellular staining, first cells were fixed and permeabilized according to the manufacturer’s instructions (Fix&Perm, Thermo Fisher) and then incubated with specific antibodies at 4°C for 30 minutes, washed, and analyzed. (A list of antibodies is provided in Supplemental Table 1.)

For live and dead cell discrimination, cell suspension was stained prior to antibody mix with 100 μL of a 1:1,000 dilution of the LIVE/DEAD Fixable Aqua Dead Cell Stain Kit (Invitrogen) in PBS at 4°C, for 30 minutes.

For intracellular staining of phosphoproteins, cell suspensions were fixed with 2% formaldehyde for 15 minutes at room temperature, washed in PBS 1x, and centrifuged. Supernatants were discarded, and cells were permeabilized in 90% methanol on ice for 30 minutes, washed twice in PBS, and thereafter, stained with specific phosphoantibodies (Supplemental Table 1) for 1 hour at room temperature. Samples were washed and resuspend in PBS 1x for flow cytometry analysis.

For proliferation, cell suspension was stained with 5 μM CFSE (Merk) at a concentration of 10^6^ cells/mL, incubated for 10 minutes at room temperature in the dark, washed 3 times in MACS buffer, and counted. Cells were then plated according to experimental conditions reported in figure legends and analyzed at flow cytometer after 4–5 days.

Samples were acquired with Novocyte 3000 (ACEA Biosciences) and LSRII Fortessa Cell Analyzer (BD Bioscience) and analyzed with Novoexpress software (ACEA Bioscience). Antibodies used are listed in the Supplemental Table 1.

### iTreg generation.

CD4^+^CD25^–^ cells were used for iTreg generation. 10^6^/mL Tconv were plated in a 24-well plate previously coated with 1 μg/mL anti-CD3 and 1 μg/mL CD28 in the presence of high concentration of IL-2 (100 U/mL) and TGF-β (5 ng/mL) in complete RPMI medium (10% FBS, glutamine, HEPES, MeOH, sodium pyruvate, and antibiotics, R10) for 24 hours at 37°C with 5% of CO_2_. Cells were then harvested, washed, and split (1 to 2) in anti-CD3/28 coated 24-well plate with IL-2 and TGF-β for additional 3 days. In some experiments, after 4 days of culture, iTregs were treated for 3 hours with pyrrophenone (10 nM) or MAFP (Methyl Arachidonyl Fluorophosphonate, 5 μM) as reported in figure legends.

### Suppression and proliferation assays.

Purified CD4^+^CD25^–^ and CD4^+^CD25^+^ or iTregs were stained with CFSE (as described in Methods, *Flow cytometry*). 1.5 × 10^5^ to 2.5 × 10^5^ cells were plated for both assays. For the suppression assays, Tconv were plated in the presence of increasing concentrations (ratio of Tconv/Treg, 1:0, 1:0.25, 1:0.5, 1:1).

To induce proliferation, cells were cultured in 200 μL R10 in 96-well plate (U-bottom wells) coated with 0.2–0.25 μg/mL anti-CD3 and anti-CD28 (eBioscience) for 3–4 days at 37°C with 5% of CO_2_ as described previously (42). Suppression of CD4^+^CD25^–^ T cell proliferation was assessed by flow cytometry as the percentage of cells in proliferation, under each condition of Treg coincubation (1:0.25, 1:0.5, 1:1), adjusted for their basal proliferation (1:0) as described previously (43).

### Mixed lymphocyte reaction.

4 × 10^5^ purified CD4^+^ T cells from allogeneic mice (CBA strain, courtesy of Cristiana Perrotta, University of Milan, Milan, Italy) were cultured with 1 × 10^5^ of purified splenic DCs and plated in 200 μL R10 in the presence of IL-2 (10 U/mL) in 96-well plate (U-bottom wells) for 5 days at 37°C with 5% of CO_2_ as described previously (44). For cytokine production, 96 hours after the culture, cells were pulsed with 0.1 μg/mL PMA and 1 μg/mL ionomycin for 4 hours at 37°C with 5% of CO_2_ in the presence of Brefeldin A (1:1,000). Cytokine analysis was performed by flow cytometry following the instructions from the fixation/permeabilization kit (Thermo Fisher).

### In vitro migration assay.

1 × 10^6^ of freshly isolated T cells were resuspended in 300 μL migration medium (RPMI 1640 supplemented with 2% fetal bovine serum) and cultured on Transwell inserts (diameter, 6.5 mm) with 5 μm pore size polycarbonate membranes. Cells were left to migrate versus migration medium, and chemokines CCL19 (200 ng/mL) or CXCL10 (300 ng/mL) placed in the bottom of the well for 3 hours. Staining for CD4, CD8, CD25, and FoxP3 was then performed before and after the migration to calculate the number of CD4^+^CD25^hi^FoxP3^+^ migrated, corrected for plated CD4^+^CD25^hi^FoxP3^+^ and expressed as a fold of migration compared with spontaneous one.

0.5 × 10^6^ iTregs were resuspended in 300 μL of migration medium as described for freshly isolated T cells. After 3 hours, the number of migrated Tregs was determined by a hemacytometer and corrected for plated iTregs, and data were expressed as fold of migration compared with spontaneous one.

### EAE induction.

Purified Tregs were intraperitoneally injected (1.4 × 10^6^ Treg/mouse) in Foxp3-reporter mice (B6.Cg-Foxp3^tm2(EGFP)Tch^/J) 16 hours before the EAE induction, and a control group received saline. EAE was induced in 8- to 10-week-old female mice by subcutaneous injection of an emulsion of MOG_35–55_ in complete Freund’s adjuvant, followed by intraperitoneal administration of pertussis toxin (500 ng) twice (at days 0 and 2). Animals were scored daily for clinical symptoms of EAE according to a 0–6 scale: 0, no clinical signs; 1, loss of tail tone; 2, tail paralysis; 3, hindlimb weakness; 4, hindlimb paralysis; 5, quadriplegia; and 6, death. At day 20, animals were euthanized by cervical dislocation and brain, and spinal cord and spleen were harvested for flow cytometry analysis. All efforts were made to minimize the number of animals used and their suffering.

### In vitro restimulation with MOG_35–55_ peptide or anti-CD3 (2C11) monoclonal antibody.

Splenocytes were plated in R10 at 2.5 × 10^6^ cell/mL density and in vitro stimulated with 50 mg/mL MOG_35–55_ peptide for 96 hours or 10 mg/mL anti-CD3 monoclonal antibody (2C11 clone) for 48 hours.

### TNBS-induced colitis model.

2 mg TNBS in 35% ethanol was administered to 8-week-old female C57BL6/J mice 7 days before and the day after the injection of 1.2 × 10^6^ CD4^+^CD25 Tregs purified from lymphoid tissues of *Srebp1c* WT and -KO female mice or vehicle (PBS). Controls consisted of mice treated with 35% ethanol. Weight changes were recorded daily; mice were euthanized on day 4, and colons were collected for cytokine quantification by ELISA.

### Seahorse metabolic analysis.

iTregs were plated on Cell-Tak-coated Seahorse Bioanalyzer XFe24 culture plates (Agilent) (0.4 × 10^6^ cells/well) for glucose stress tests and ATP rate assay. For analysis of glycolysis, iTregs were cultured in assay media consisting of DMEM, pH 7.4, supplemented with glutamine (2 mM) and glucose (10 mM); oligomycin (1.5 μM) and 2-DG (50 mM) were added according to the glucose stress test. For ATP production, iTregs were cultured in DMEM, pH 7.4, supplemented with 10 mM glucose, 1 mM pyruvate, and 2 mM glutamine; oligomycin (1.5 μM) and rotenone/antimycin A (0.5 μM) were added according to Real-Time ATP Rate assay (Agilent). For all tests, drugs were injected in a Seahorse analyzer (Agilent), and OCR and ECAR data were analyzed through the Wave software (Agilent). A list of reagents is provided in Supplemental Table 3.

### Adenosine quantification.

Adenosine was quantified using a commercial fluorometric kit (Cell Biolabs) according to the manufacturer’s instructions. Supernatants from iTreg cultures, pretreated or not with pyrrophenone, were used for analysis (Supplemental Table 3).

### Metabolomics.

iTregs (2 × 10^6^) (for steady-state metabolomics) or iTregs supplemented with D-Glucose-1,2-^13^C_2_ (10 μM for 24 h, for metabolic fluxes) were pelleted and snap frozen in dry ice and stored at −80°C until analysis and run on an API-4000 triple quadrupole mass spectrometer (AB Sciex) coupled with a HPLC system (Agilent).

Detailed methodology is described in the Supplemental Methods.

### Liquid chromatography–electrospray ionization tandem mass spectrometry.

iTreg (5 × 10^6^) pellets were snap frozen in dry ice and stored at −80°C until analysis. Lipid species were analyzed by liquid chromatography–electrospray ionization tandem mass spectrometry on a Nexera X2 UHPLC system (Shimadzu) coupled with a hybrid triple-quadrupole/linear ion trap mass spectrometer (6500+ QTRAP system; AB SCIEX, The Netherlands).

Detailed methodology is described in the Supplemental Methods.

### RNA-seq.

iTreg (5 × 10^6^) pellets were snap frozen in dry ice and stored at −80°C until analysis. Total RNA from iTregs was extracted using the Monarch total RNA miniprep kit (New England Biolabs) according to manufacturer’s instructions. Sequencing was performed on the Illumina NextSeq 550 using the NextSeq 500/550 High Output Kit v2.5 (Illumina).

Detailed methodology is described in the Supplemental Methods.

### Western blotting.

Frozen cell pellets from iTregs (2 × 10^6^ to 5 × 10^6^ cells) were resuspended into 60–100 μL RIPA buffer (50 mM Tris, pH 8.0, 150 mM NaCl, 0.1% sodium dodecyl sulphate, 1.0% NP-40, 0.5% sodium deoxycholate) with 0.6 μL proteinase/phosphatase inhibitor (100X), left to homogenize 15 minutes in ice, and then vortexed again followed by a second incubation of 15 minutes in ice. Samples were then centrifuged, and the supernatants were collected into new vials. Protein content was assessed by the in-house Lowry Protein Assay. The same amount of proteins (between 15 and 40 μg/mL) per sample, previously denatured by the addition of Laemmli buffer and β-mercaptoethanol and incubation for 5 minutes at 100°C, were loaded on a polyacrylamide gel and thereafter transferred to a nitrocellulose sheet. Unspecific binding sites were saturated after 1-hour incubation in agitation with 5% nonfat milk or 5% BSA in TBS-T 1X depending on the specificities of the primary antibody. Incubation with primary antibodies was performed overnight at 4°C in agitation. Antibodies are listed in Supplemental Table 2. Membranes were then incubated with secondary antibodies conjugated to the horseradish peroxidase for 1 hour at room temperature and acquired to Odyssey Imagers (LICORbio). Densiometric analysis of the blots was performed using Image Studio Lite version 3.1 (LI-COR) program, and the intensities of the target proteins were normalized to the respective value of β-actin, used as housekeeping gene.

### Statistics.

GraphPad Prism 9 and Excel were used for graphical presentation and statistical analysis. Results are presented as mean per group ± SEM, and statistical analysis was performed using a nonparametric test, multiple 2-tailed *t* tests, or 1- or 2-way ANOVA with a 95% CI, according to that reported in figure legends.

For RNA-seq analysis, *P* values were corrected for multiple testing using the Benjamini-Hochberg false discovery rate. DEGs were taken into consideration based on a log_2_-fold change <−0.5 and >0.5 and an adjusted *P* value < 0.05. For downstream analysis, Gene ontology (GO) using the Database for Annotation, Visualization, and Integrated Discovery platform (DAVID, https://davidbioinformatics.nih.gov/; National Institute of Allergy and Infectious Diseases) and IPA (QIAGEN) were used.

### Study approval.

All the procedures were performed according to the principles set out in the following laws and policies governing the care and use of laboratory animals: Italian government law (D.lgs 26/2014; authorization n.19/2008-A, issued March 6, 2008, by the Ministry of Health); the NIH *Guide for the Care and Use of Laboratory Animals* (National Academies Press, 2011); and European Union directives and guidelines (EEC Council Directive 2010/63/UE). Authorization for animal use has been obtained from the Italian Ministry of Health (Progetto di Ricerca 579/2015, 271/2020, 698/2023). All efforts were made to minimize animal suffering and to use the fewest animals.

### Data availability.

Data are available in the Supporting Data Values file. The RNA-seq dataset has been deposited in the Sequence Read Archive data of the NIH (https://www.ncbi.nlm.nih.gov/sra) with the accession code PRJNA1167636. The raw data underlying this article will be shared on reasonable request to the corresponding author.

## Author contributions

Conceived and designed the experiments: FB, CP, SP, NM, G Matarese, and GDN. Performed the experiments: FB, CP, SP, MI, A Moregola, A Moretti, JB, CR, GDR, and CLR. Analyzed the data: FB, CP, SP, MS, JB, GBV, FG, and GDN. Contributed to discussion of results: FB, CP, SP, JB, G Mondanelli, MG, NM, G Matarese, and GDN. Wrote the first draft of the manuscript: FB and GDN. Reviewed and edited the manuscript: FB, CP, SP, JB, NM, G Matarese, and GDN.

## Conflict of interest

The authors have declared that no conflict of interest exists.

## Funding support

- Fondazione Cariplo (grant no. 2019-1560) to FB.
- University of Milan (grant no. PSR2022_DIP_022_AZIONE_A_FBONA) to FB.
- Progetti di Rilevante Interesse Nazionale (PRIN) 2022 from the Italian Ministry of University and Research (MUR) (grant no. 2022NBKCWP) to FB.
- Progetti di Rilevante Interesse Nazionale (PRIN) (2017 K55HLC from the Italian Ministry of University and Research [MUR]) to GDN.
- Ministry of Health Ricerca Finalizzata (RF-2019-12370896) to GDN.
- CARDINNOV, Ministry of Research and University under the umbrella of the Partnership Fostering a European Research Area for Health (ERA4Health) (GA no. 101095426 of the EU Horizon Europe Research and Innovation Programme to GDN).
- Horizon Europe-European Research Executive Agency HORIZON-MSCA-2024-DN-01 Understanding Lipid ImmunoMetabolIsm To trEat Disease (UNLIMITED) to GDN.
- 4EU+ Alliance, SEED4EU+ 2025 “SCAMEDI,” to GDN.
- Fondazione Cariplo (2014-0991) to NM.
- Italian Ministry of Health with Ricerca Corrente and “5xmille” funds to NM.
- Fondazione Italiana Sclerosi Multipla (FISM) (2022-PR-Single/013) to CP.
- Italian Ministry of Health PNRR M6/C2_CALL 2022 (PNRR-MAD-2022-12376126) to CP.
- PNRR M6/C2_CALL 2023 (PNRR-MCNT2-2023-12377866).
- Italian Ministry of University and Research (MUR) (NRRP)-PRIN (P2022T4PKT) to CP.
- Italian Ministry of University and Research (MUR) (NRRP)-PRIN, (2022LNHZAP) to G Matarese.
- Italian Ministry of Health M6/C2_CALL 2022 (PNRR-MAD-2022-12375634) to G Matarese.
- PNRR M6/C2_CALL 2023 (PNRR-MCNT2–2023-12378040).
- MUR/PNRR Extended Partnership (INF-ACT no. PE00000007 and MNESYS no. PE00000006) to G Matarese.
- MUR Progetto Eccellenza (2022–2027) to the Dipartimento di Scienze Farmacologiche e Biomolecolari “Rodolfo Paoletti,” Università degli Studi di Milano.

## Supplementary Material

Supplemental data

Unedited blot and gel images

Supporting data values

## Version 1 07/21/2026

In-Press Preview

## Version 2 09/01/2026

Electronic publication

## Version 3 09/09/2026

Corrected affiliation no. 5
