# Supplementary material for: SREBP1c modulates Treg immunobiology through a phospholipid-dependent adenosine pathway
Source: J Clin Invest. 2026 Jul 21;136(17):e201325. doi: 10.1172/JCI201325 (PMC13528931; doi:10.1172/JCI201325)
Supplement: Supplementary file 1 [file jci-136-201325-s007.pdf]

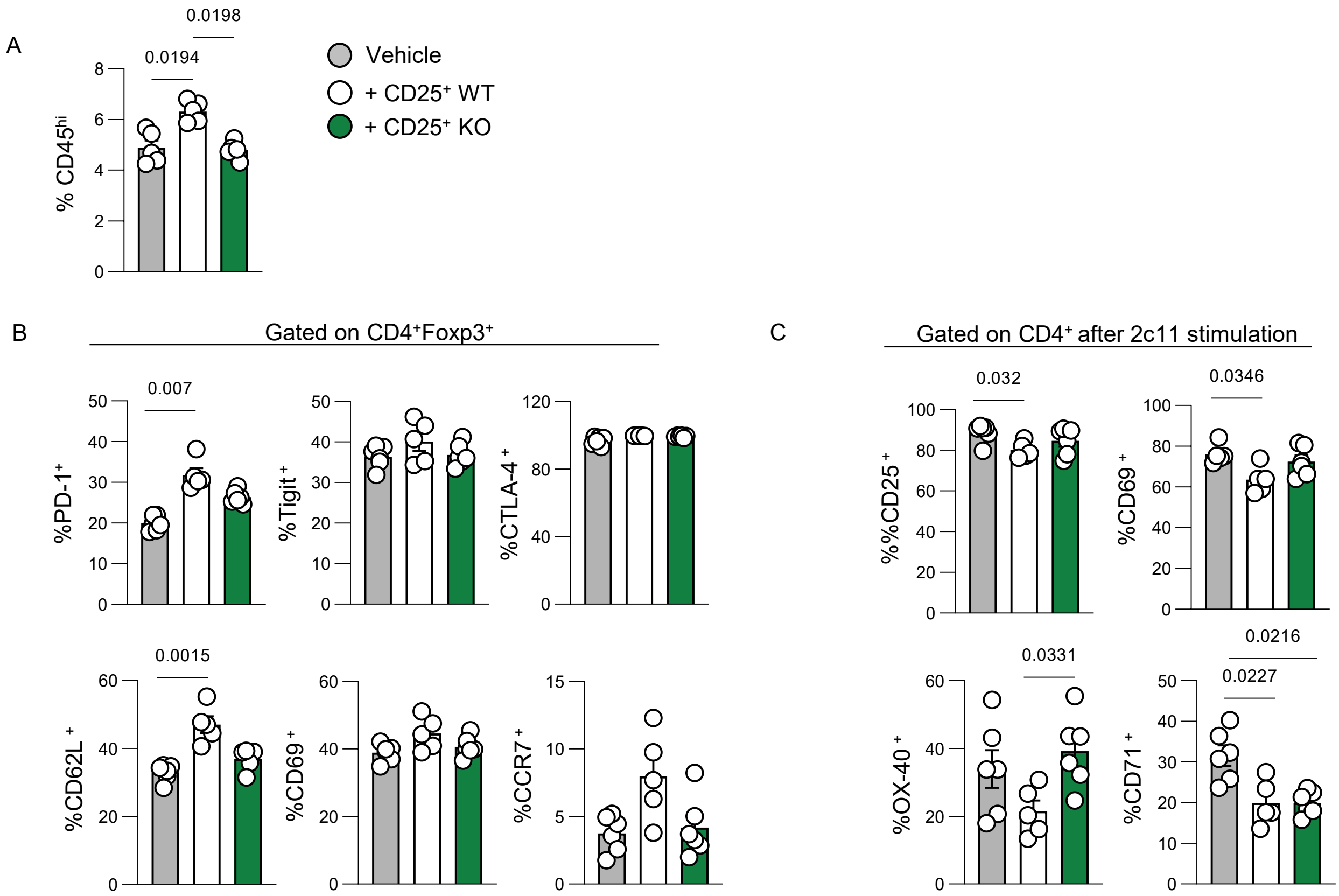

Supplementary Figure 1

A-B) Percentage of $GFP^+/CD4^+$ Tregs (A) and expression of Treg-specific markers (PD-1, Tigit, CTLA-4, CD62L, CD69, CCR7) in $GFP^+/CD4^+$ cells (B) from splenocytes isolated from EAE mice 20 days after MOG35-55 immunization. C) Expression of activation markers (CD25, CD69, OX-40, CD71) in $CD4^+$ T cells in splenocytes isolated from EAE mice and in vitro stimulated with 2C11 (polyclonal stimulation), 20 days after MOG35-55 immunization. N=4-10/ group; data are presented as mean +/- SEM. Statistical analysis has been performed with one- way Anova Kruskal Wallis test (A-C).

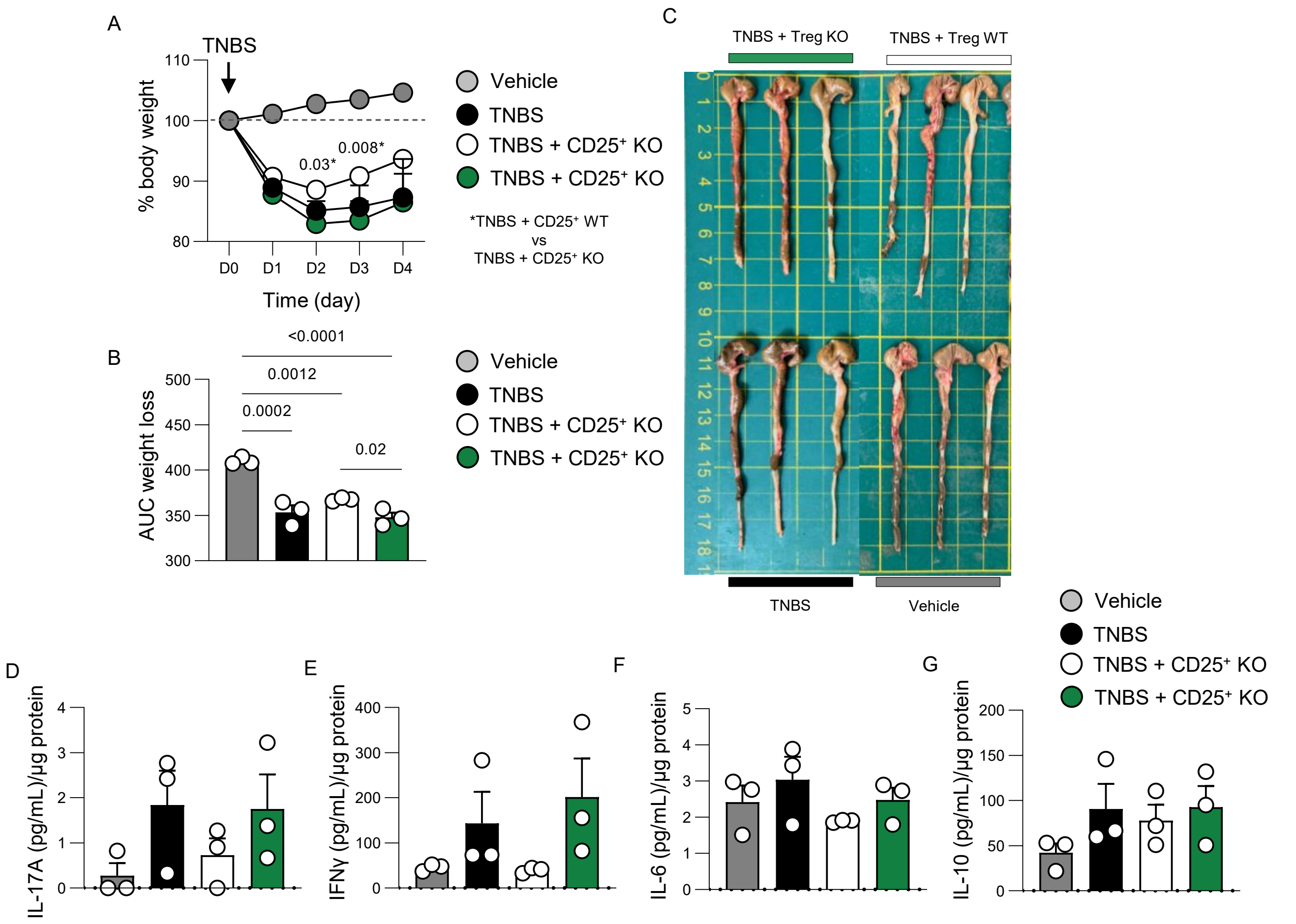

Supplementary Figure 2

A) Percentage of body weight reduction in mice undergoing TNBS-induced colitis with or without the administration of WT or KO Tregs, compared to control mice receiving vehicle. Body weight was taken before TNBS administration (day 0) and for the following 5 days; B) area under the curve of the percentage of body weight reduction; C) representative pictures of colons from experimental groups after 5 days from TNBS administration; D-G) Quantification of pro-inflammatory cytokines (IL-17A, IFNγ, IL-6) and anti-inflammatory (IL-10) from the colons of experimental mice. N=3/group; data are shown as mean +/- SEM. Statistical analysis was performed with one-way Anova (A-B, D-G).

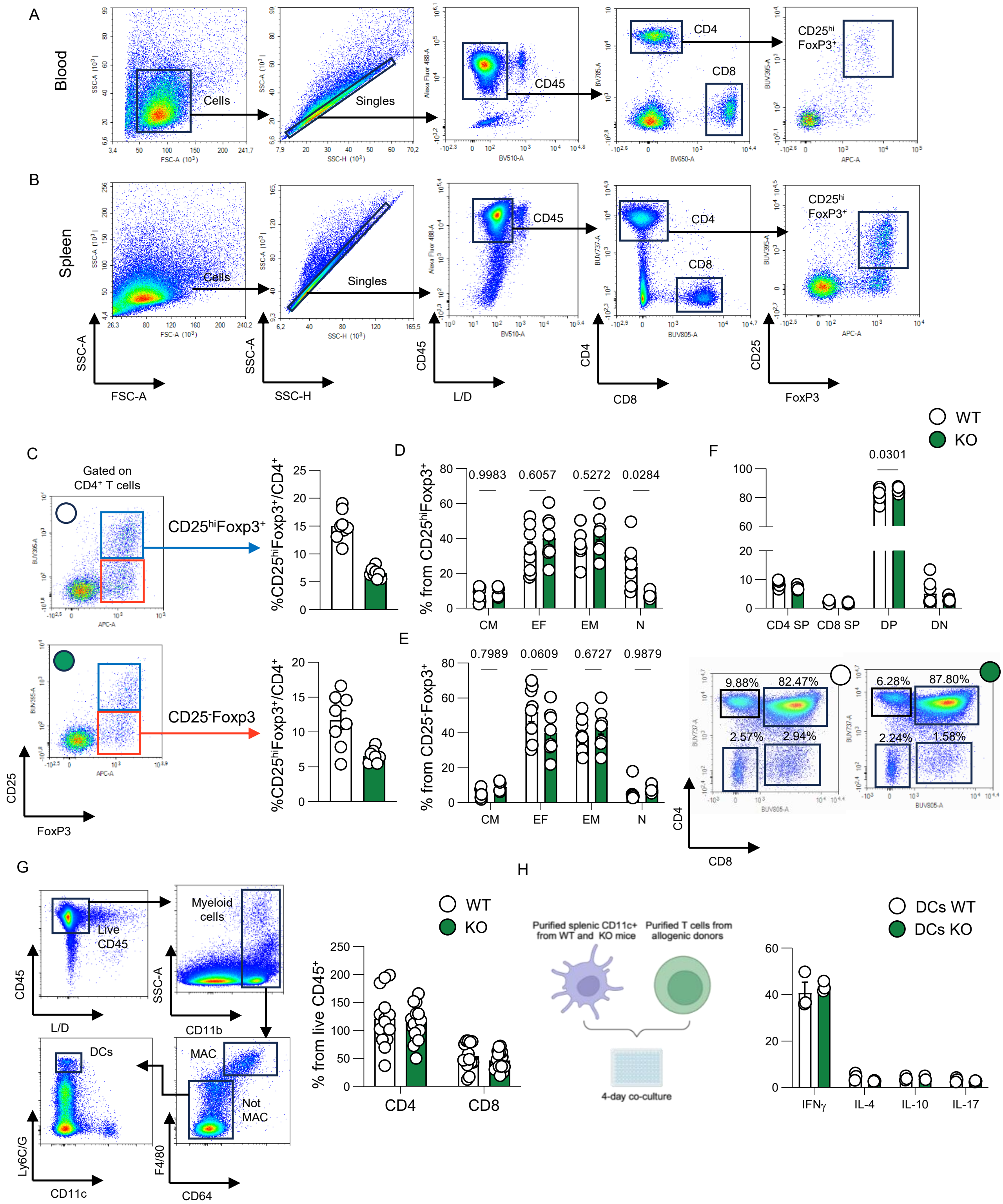

Supplementary Figure 3

A-B) Gating strategy for Treg identification by flow cytometry in the circulation (A) and spleens (B) of *Srebp1c* KO and WT mice. Cells were gated according to dimension based on FSC-A and SSC-A, singles discriminated by SSC-A vs SSC-H gating and within this population live leukocytes identified as CD45+ and live/dead staining (L/D) negative. Helper and cytotoxic T cells were identified by the expression of CD4 and CD8 markers,

respectively, and within CD4 T cells, Tregs were identified as CD25hi FoxP3+. Frequency of CD4 and CD8 single and double positive or negative cells. Representative density plots from flow cytometry analysis are reported. C) Representative plots and frequency of CD25hiFoxP3+ and CD25-FoxP3+ Tregs in the spleen from *Srebp1c* WT and KO mice. D-E) Frequency of central memory (CD62L+CD44+, CM), effector (CD62L-CD44-, EF), effector memory (CD62L-CD44+, EM), and naïve (CD62L+CD44-, N) within CD25hiFoxP3+ (D) and CD25-FoxP3+ (E) Tregs in the spleen of *Srebp1c* WT and KO mice. F) Frequency of CD4 and CD8 single and double positive or negative cells. Representative density plots from flow cytometry analysis are reported. G) Gating strategy for dendritic cells (DCs) and macrophages (Mac) identification in the spleens of *Srebp1c* KO and WT mice. Live leukocytes were identified as CD45+ and L/D- cells, and within this population, myeloid cells were distinguished by CD11 b expression. Within CD11b+, macrophages were identified by the expression of F4/80 and CD64 (Mac), while in the non-Mac population, DCs were gated as CD11c+ Ly6C/G- cells. Percentage of DCs and Mac out of live leukocytes (CD45) from the spleens of *Srebp1c* KO and WT mice. H) Schematic representation of a mixed lymphocyte reaction between DCs, purified as CD11c+ from the spleen of *Srebp1c* KO and WT mice, and T cells isolated from the spleens of allogenic donors (CBA mice) cultured for 4 days. Cytokine production (IFNγ, IL-4, IL-10 and IL-17) was measured by flow cytometry after re-stimulation with PMA/ionomycin in the presence of brefeldin (4 h). N=3-8/ group; data are presented as mean +/- SEM. Statistical analysis was performed with two-way Anova (C, D, E) and unpaired non-parametric T test (F, G).

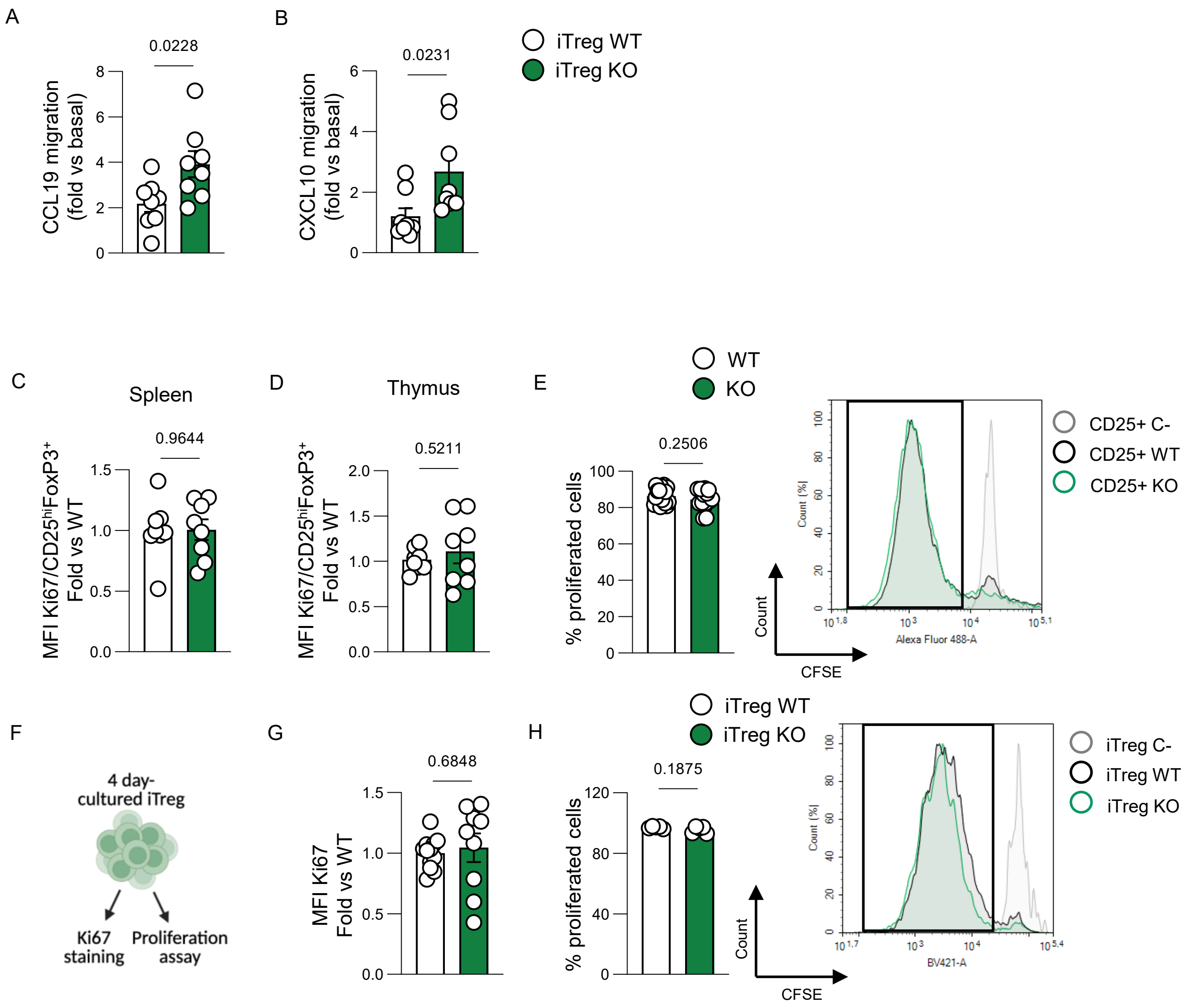

Supplementary Figure 4

A-B) In vitro migration assay of in vitro generated Tregs (iTregs) from WT and *Srebp1c* KO mice toward CCL19 (A) and CXCL10 (B) chemokines after 3 hours of incubation in 24-well plate transwells. Results are corrected versus spontaneous (basal) migration toward the medium. C-D) Mean fluorescence intensity (MFI) of Ki67 in CD25hiFoxP3+/CD4+ cells from the spleen (C) and thymus (D) of *Srebp1c* WT and KO mice. Values are corrected for WT values and represent two independent experiments (n=4/group). E) Proliferation of freshly isolated CD4+CD25+ Tregs by CFSE dilution. 0,15 x 10^6 cells were cultured for 4 days in anti-CD3/CD28 (0.25 μg/mL) coated 96-well plate. Proliferation was measured by flow cytometry and quantified as the percentage of cells with reduced CFSE fluorescence (black square) relative to the unstimulated negative control (C-). Values represent three independent experiments (n=4-5/group). F) Graphic representation of the experimental approaches used to test iTreg proliferation. G) Mean fluorescence intensity (MFI) of Ki67 in *Srebp1c* WT and KO iTregs. Data are corrected on WT values. H) Proliferation of iTregs by TraceViolet dilution. 0,15 x 10^6 cells were cultured for 4 days in anti-CD3/CD28 (0.25 μg/mL) coated 96-well plate. Proliferation was measured by flow cytometry and quantified as the percentage of cells with reduced TraceViolet fluorescence (black square)

relative to the unstimulated negative control (C-). N=4-12/group; data are presented as mean +/- SEM. Statistical analysis was performed with unpaired non-parametric T test (A-E, G, H).

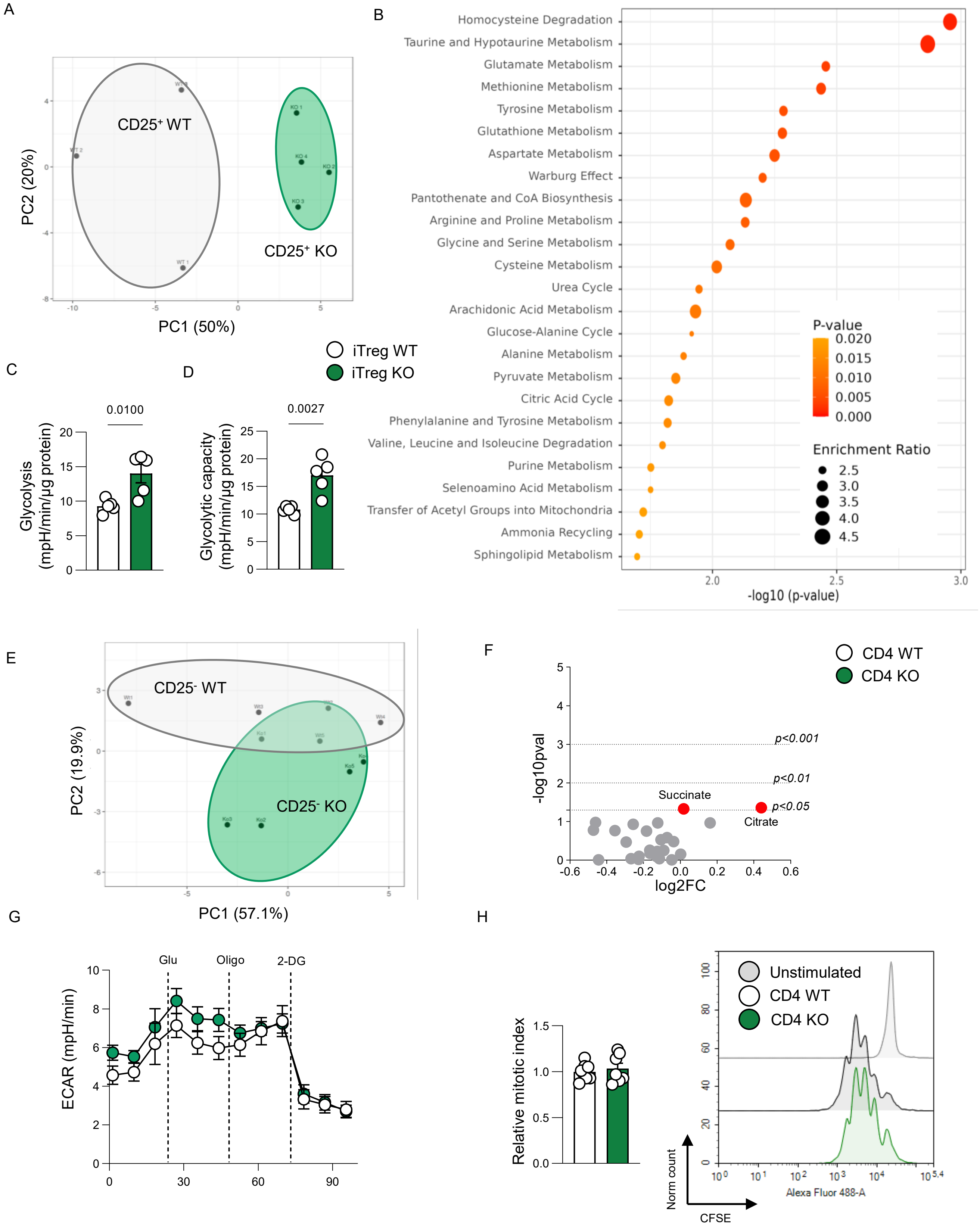

Supplementary Figure 5

A) PCA analysis from targeted metabolomics of Srebp1c WT and KO CD25+ Tregs. B) Top 25 significant pathways (-log10 p-value>1.3) from Metabolite Set Enrichment Analysis (MSEA) of significantly different

metabolites performed by MetaboAnalyst® from targeted metabolomics of WT and *Srebp1c* KO CD25+ Tregs. C-D) Glycolysis (C) and Glycolytic capacity (D) measured by Glucose stress test tested by Seahorse® on iTregs from *Srebp1c* WT and KO mice. E) PCA analysis from targeted metabolomics of Srebp1c WT and KO CD4+ CD25- T conventional cells. F) Volcano plot from targeted metabolomics of Srebp1c WT and KO CD4+ CD25- T conventional cells. G) ECAR analysis from glucose stress test tested by Seahorse on Srebp1c WT and KO CD4+ CD25- T conventional cells. H) Proliferation of CD4 T cells after CD3/CD28 stimulation for 4 days. Data are presented as relative mitotic index compared to WT cells. N=3-8/ group; gata are presented as mean +/- SEM. Statistical analysis was performed with unpaired non-parametric T test (C, D, H).

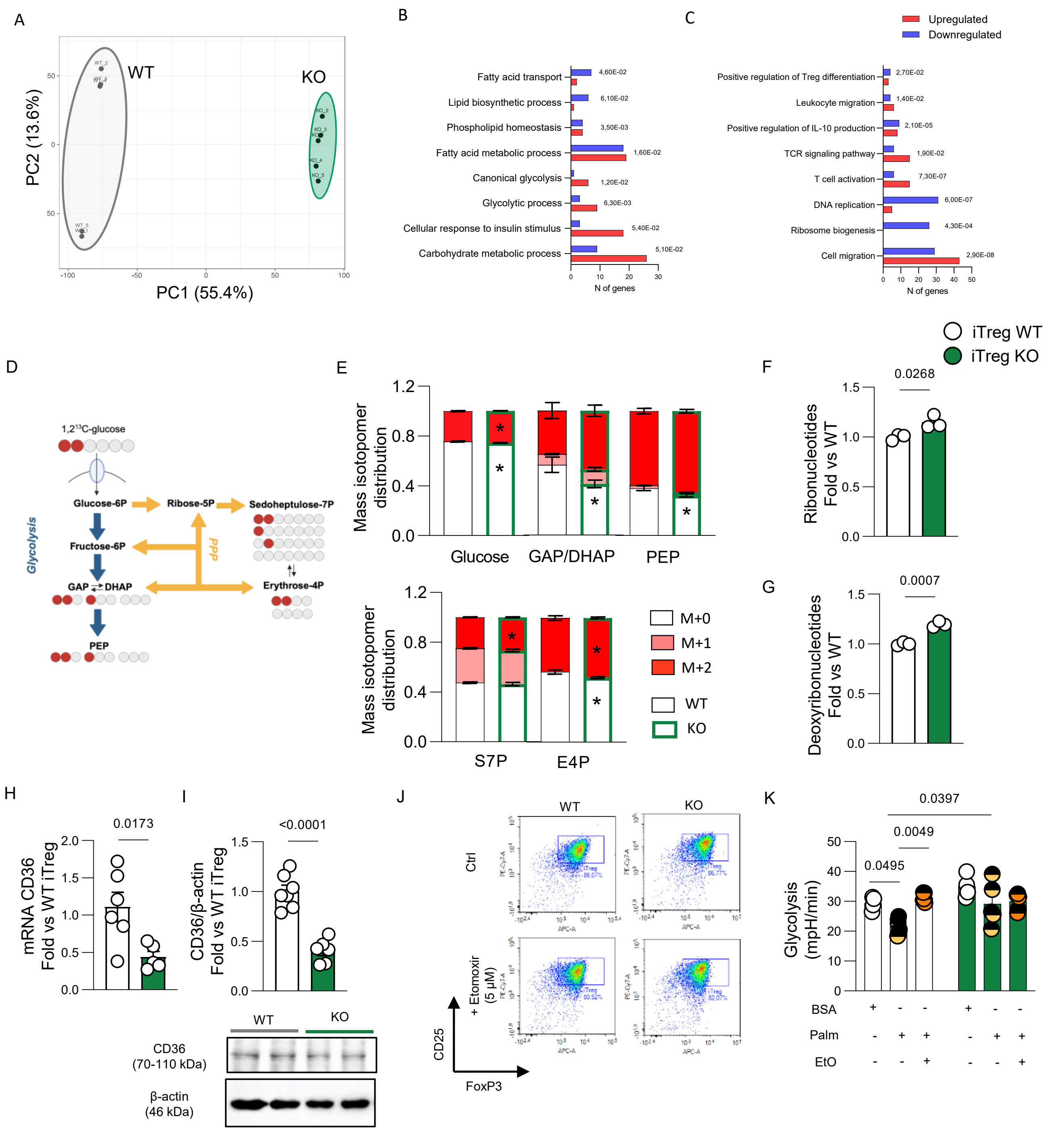

Supplementary Figure 6

A) Principal component analysis (PCA) from RNA-seq of *Srebp1c* WT and KO iTregs. B-C) Gene ontology (GO) terms of Biological Processes (BP) from David database analysis of DEGs from RNA-seq of *Srebp1c* KO vs WT iTregs, showing the number of upregulated (red) and downregulated (blue) genes for each pathway and its pValue. Histograms show the most affected pathways related to metabolism and energy (B) and to Treg function and activation (C). D) Graphic representation of labeled metabolites of glycolytic and PPP pathway detected after incubation of iTregs for 2h with DMEM medium reconstituted with 5mM of “cold” glucose and 5mM of [1,2-

C13] D-glucose. E) Mass topoisomer distribution (1, M1, or 2, M2, labeled carbons) after analysis of 1,2C-glucose fluxes of Glucose, Dihydroxyacetone phosphate/Glyceraldehyde 3-phosphate (DHAP/GAP), phosphoenolpyruvate (PEP), erithrose-4-phosphate (E4P), sedoheptulose-7-phosphate (S7P) in *Srebp1c* KO vs WT iTregs. F-G) Sum of ribonucleotides (J) and deoxyribonucleotides (K) normalized on WT measured by metabolomics of Srebp1c KO and WT iTregs. H-I) Gene expression by RT-qPCR (D) and protein quantification by western blot analysis (E) of CD36 in WT and *Srebp1c* KO iTregs; representative pictures from western blot analysis for CD36 are presented. J) Representative density plots of CD25 and FoxP3 expression from flow cytometry analysis of *Srebp1c* WT and KO iTregs in the presence or absence of etomoxir (5 μM for 4 hours). K) Glycolysis (mpH/min) analyzed by Seahorse® Glucose Stress Test assay tested in iTregs from WT and *Srebp1c* KO pre-incubated with BSA, palmitate (100 μM for 30 minutes) or palmitate + etomoxir (5 μM for 4 hours). N=5 biological replicates/ group (A-C), 3-7 (E-I, K); data are presented as mean +/- SEM and statistical was performed with unpaired non-parametric Ttest (E-I) and two-way Anova (K).

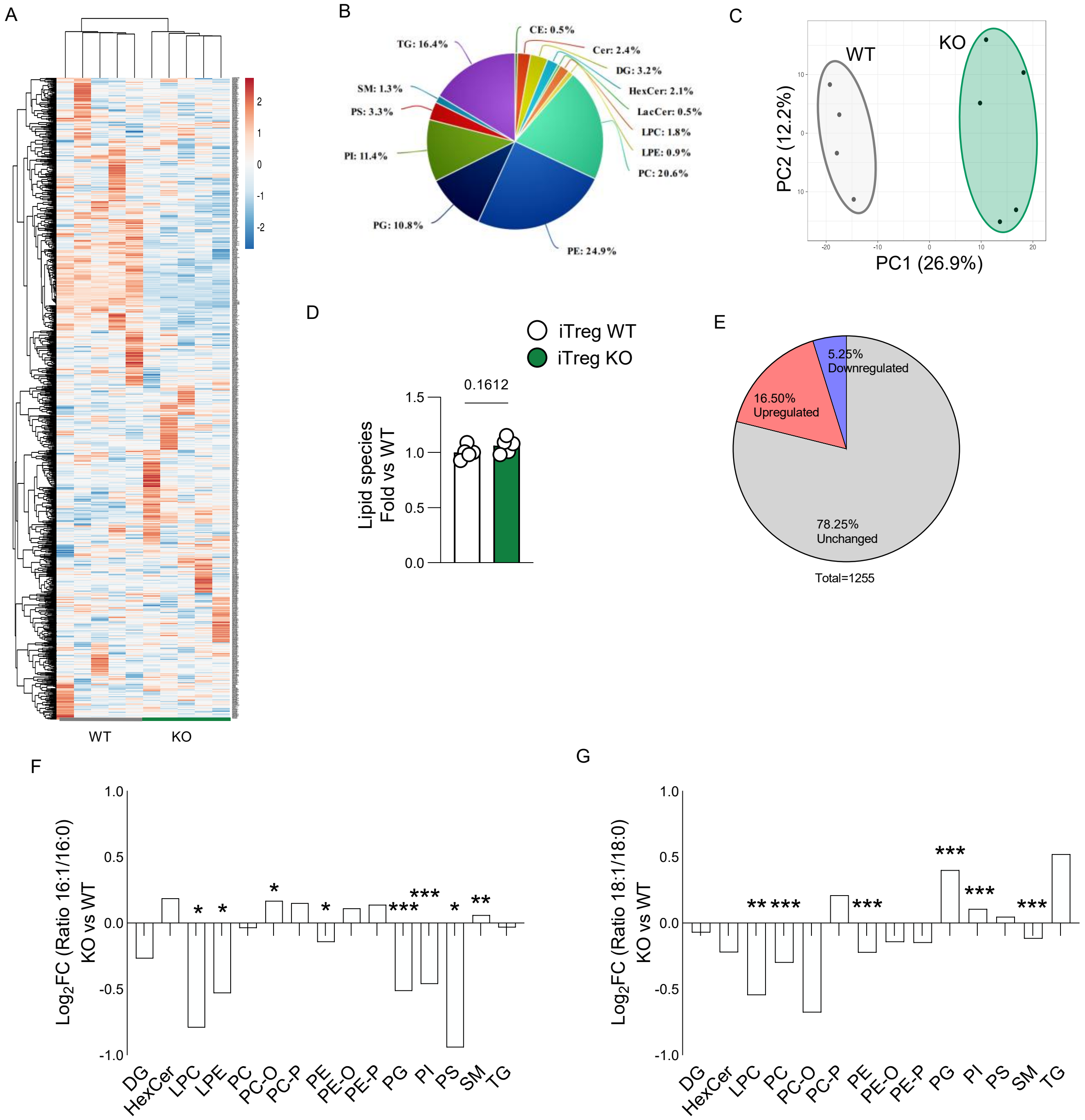

Supplementary Figure 7

A) Heatmap from lipid species analysis from lipidomics of *Srebp1c* KO and WT iTregs. B) Most abundant lipid classes identified by lipidomic analysis of iTregs from WT and *Srebp1c* KO mice. C) PCA analysis from lipidomic analysis of iTregs from WT and *Srebp1c* KO mice (5 biological replicates/group). D) Sum of all lipid species detected by lipidomics in Srebp1c WT and Ko iTregs, normalized on WT mean. E) Distribution of significantly increased lipid species (16,50%) and decreased (5,22%) on total lipid species identified (1255). F-G) Log_2FC of the ratio between 16:1/16:0 (F) and 18:1/18:0 (G) of the main lipid classes identified by lipidomics (DG, HexCer, LPC, LPE, PC, PC-O, PC-P, PE, PE-O, PE-P, PG, PI, PS, SM and TG) in *Srebp1c* KO iTregs compared to WT.

Lipidomics was performed on 5 biological replicates/group. In panel D data are presented as mean +/- SEM and statistical analysis was performed with unpaired non-parametric T test.

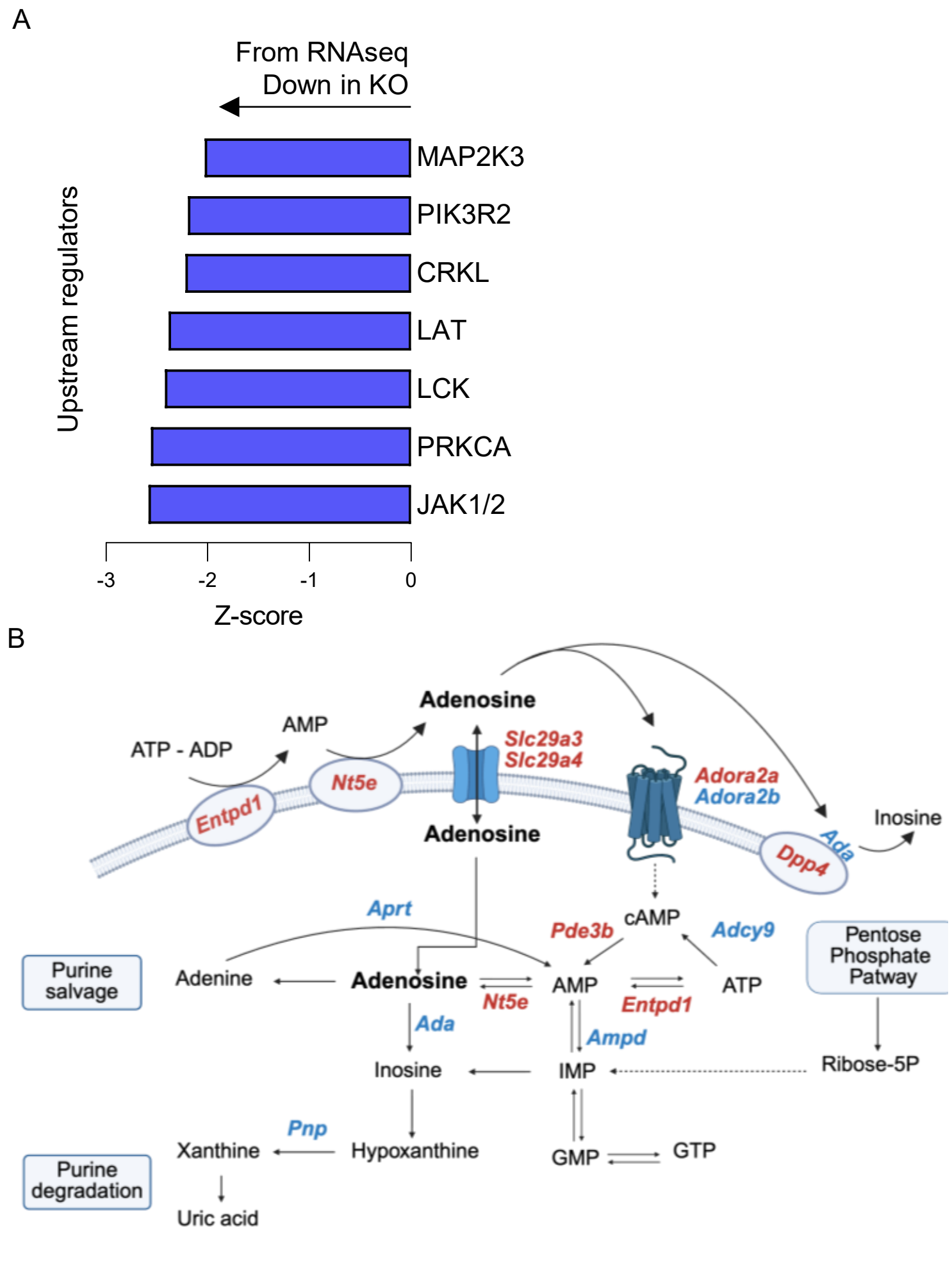

Supplementary Figure 8

A) Upstream regulators associated with intracellular signaling and cytokine pathways predicted down-regulated (negative z-score, blue) by IPA® software from RNA-seq of WT vs Srebp1c KO iTregs. B) Graphic representation of adenosine metabolism consisting of extracellular ectonucleotidases, transporters, intracellular metabolic enzymes and adenosine receptors that together control its production, uptake and intracellular signaling. In more details, Nt5e (CD73) converts AMP to adenosine; Entpd1 (CD39) hydrolyzes ATP and ADP to AMP, supplying substrate for CD73; Dpp4 (CD26) binds ADA and modulates extracellular adenosine clearance; Pnp cleaves purine nucleosides; Ampd1/2/3 deaminate AMP to IMP; Adk phosphorylates adenosine to AMP; Ada deaminates adenosine to inosine; Slc29a4 (ENT4) mediates bidirectional, pH-sensitive uptake of adenosine; Slc28a2 (CNT2) imports adenosine into cells; Pde3b hydrolyzes cAMP to AMP; Adcy9 generates intracellular cAMP from ATP downstream of Gs signaling; Adora2a is a high-affinity Gs-coupled extracellular adenosine receptor; Adora2b is a lower-affinity Gs-coupled

extracellular adenosine receptor activated at higher adenosine concentrations. C) Expression of genes related to adenosine synthesis, catabolism, transport, and signaling from RNA-seq on *Srebp1c* KO and WT iTregs. Dimension of the bubble represents the $-\log_{10}$ pValue, while the color represents the $\log_2$FC compared to WT (in blue downregulated and in red upregulated genes). RNA-seq was performed on 5 biological replicates/group.

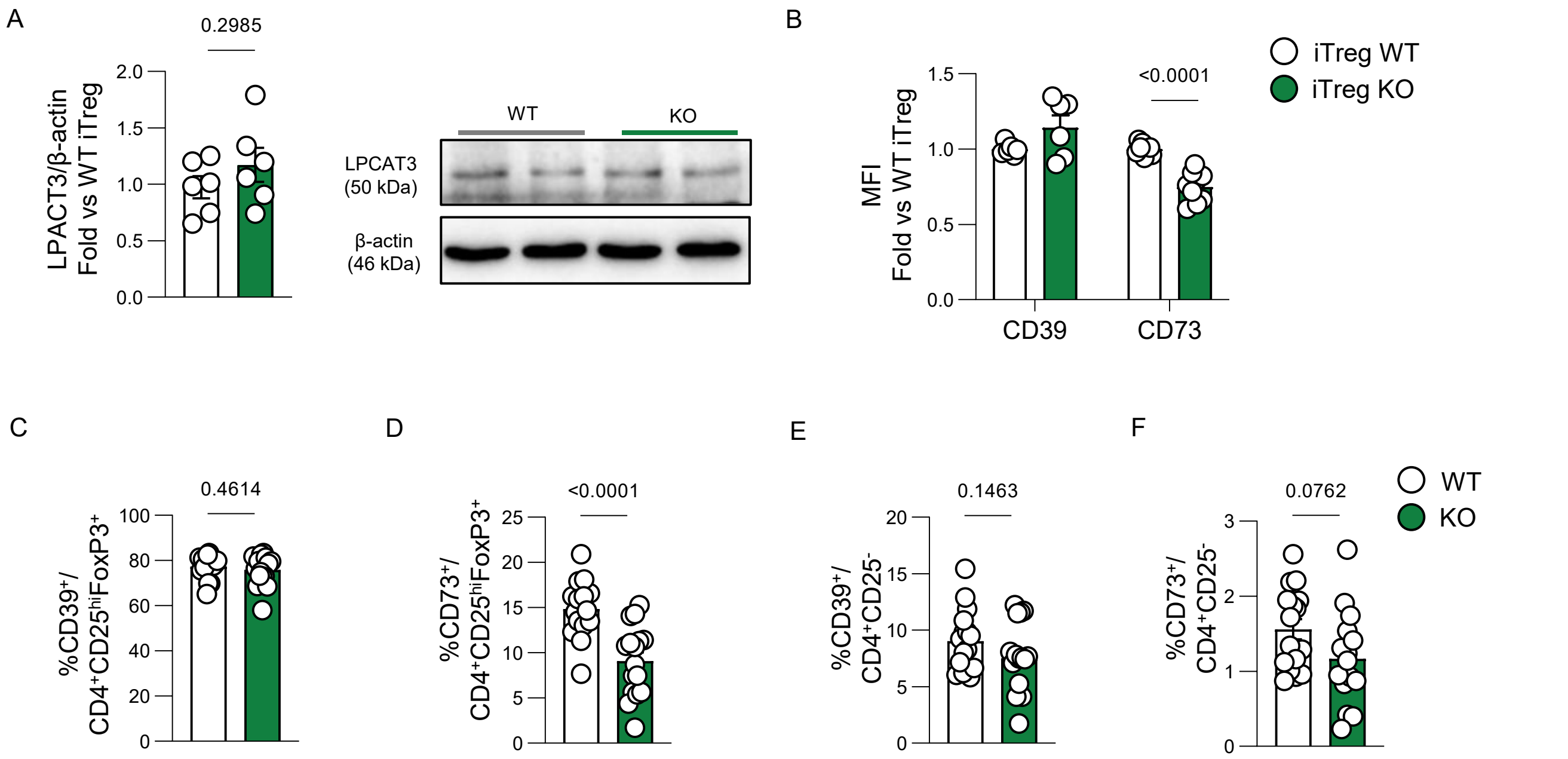

Supplementary Figure 9

A) Quantification of LPCAT3 by Western Blot analysis of *Srebp1c* WT and KO iTreg. Data are corrected on β-actin abundance; a representative western blot for LPCAT3 and β-actin is shown. B) Expression of CD39 and CD73 by flow cytometry in *Srebp1c* WT and KO iTregs. Data are expressed as a fold change of mean intensity fluorescence (MFI) compared to WT. C-D) Expression of CD39 (C) and CD73 (D) by flow cytometry in CD4+CD25hi FoxP3+ Tregs from the spleen of *Srebp1c* WT and KO mice. E-F) Expression of CD39 (E) and CD73 (F) by flow cytometry in CD4+CD25- T conventional cells from the spleen of *Srebp1c* WT and KO mice. N=5-16/ group; data are presented as mean +/- SEM. Statistical analysis was performed with unpaired non-parametric T test (A, B-F).

Supplementary Table 1.
List of antibodies for flow cytometry analysis

ANTIGEN	FLUOROPHORE	CLONE	DILUTION	BRAND & CATALOG NUMBER
Anti-mouse CD4	BV786	RM4-5	1:100	BD Bioscience Cat#563727
Anti-mouse CD8	BV650	53-6.7	1:100	BD Bioscience Cat#563234
Anti-mouse CD25	BUV395	PC61	1:100	BD Bioscience Cat#740879
Anti-mouse CD25	PECy7	PC61-5	1:100	ThermoFisher Cat#25-0251-81/82
Anti-mouse Foxp3	APC	FJK-16S	1:100	ThermoFisher Cat#17-5773-82
Anti-mouse CD62L	BV605	MEL-14	1:100	BD Bioscience Cat#56-3252
Anti-mouse CD44	Af700	IM7	1:100	Invitrogen, Cat#56-0441-82
Anti-mouse CD69	APC-Cy7	HI.2F3	1:100	BD Bioscience Cat#561240
Anti-mouse CD71	BV421	RI7217	1:100	Biolegend Cat#113813
Anti-mouse OX40	BV786	OX-86	1:100	BD Bioscience Cat#740945
Anti-Hmouse Helios	PE	22F6	1:100	BD Bioscience Cat#563801
Anti-mouse GITR	PercP Cy 5.5	DTA-1	1:100	Biolegend Cat#126316
Anti-mouse Ki67	Alexa Fluor 488	SolA15	1:100	ThermoFisher Cat#53-5689-80
Anti-mouse CD152 (CTLA-4)	PECF594	UC10-4F10-11	1:100	BD Bioscience Cat#564332
Anti- mouse CD279 (PD-1)	BV510	J43	1:100	BD Bioscience Cat#744544
Anti-mouse CD73	Alexa Fluor488	TY/23	1:100	BD Bioscience Cat#561545
Anti-mouse CD39	PeCy7	Duha59	1:100	Biolegend Cat#143805
Anti-mouse CCR2	BV650	475301	1:100	BD Bioscience Cat#747968
Anti-mouse CCR3	BV480	83103	1:100	BD Bioscience Cat#747817
Anti-mouse CD195 (CCR5)	BV650	C34-3448	1:100	BD Bioscience Cat#743698
Anti-mouse CD197 (CCR7)	BV421	4B12	1:100	BD Bioscience Cat#562675
Anti-mouse CD183 (CXCR3)	BV711	CXCR3-173	1:100	BD Bioscience Cat#740825
Anti-mouse RorγT	PE	B2D	1:100	Invitrogen, Cat#12-6981-82
Anti-mouse Tbet	BV421	O4-46	1:100	BD Bioscience Cat#563318
Anti-mouse GATA3	BB700	L50-823	1:100	BD Bioscience Cat#566643
Anti-mouse CD45	BUV563	30-F11	1:100	BD Bioscience Cat#612924
Anti-mouse CD11b	Af700	M1/70	1:100	Biolegend Cat#101222
Anti-mouse CD64	PE/DAZZLE	X54-5/7.1	1:100	Biolegend Cat#139319 / 139320
Anti-mouse CD11c	BV786	HL3	1:100	BD Bioscience Cat#563735
Anti-mouse Ly6C/G	BV605	RB6-8C5	1:100	BD Bioscience Cat#563299
L/D Aqua	BV510	/	1:1000	Invitrogen Cat#L34957A
Anti-mouse CD3	purified	500A2	/	Biolegend, #152302
Anti-mouse CD28	purified	37.51	/	Biolegend, #102102

Supplementary Table 2.
List of antibodies for western blot analysis

TARGET	MOLECULAR WEIGHT	BRAND	CATALOG NUMBER	DILUTION
AKT	60 kDa	Cell signaling	9272s	1 : 1000
Phospho-Akt (Ser473) (587F11)	60 kDa	Cell signaling	4051L	1 : 1000
CD36	70-110 kDa	Cell signaling	868255F	1 : 1000
pcPLA2	95 kDa	Cell signaling	2831S	1:1000
LPCAT3	50 kDa	Protein tech	67882-1-IG	1:1000
β actin	46 kDa	Merck	A5441	1:5000

Supplementary Table 3.
List of reagents

REAGENT	BRAND	CATALOG NUMBER
1X RBC Lysis Buffer	(eBiosceince) Thermofisher	00-4333-57
Fix&Perm	Thermofisher	00-5333
EasySep™ Kit	StemCell Technology	18782
CFSE	Merk	21888
Human rIL-2	(Peprotech) Thermofisher	200-02
Human rTGFβ	(Peprotech) Thermofisher	100-21
Human rCCL19	(Peprotech) Thermofisher	250-27B
Human rCCL21	(Peprotech) Thermofisher	250-16
RPMI	Euroclone	ECB2000
BD GolgiPlug™	BD biosciences	555029
Transwell™ inserts	Costar	CLS3421-48EA
DMEM	Agilent	103575-100
Glutamine	Agilent	103579-100
Glucose	Agilent	103577-100

Supplementary Table 3.
List of reagents

COMPOUND NAME	TARGET	BRAND	CATALOG NUMBER	WORKING CONCENTRATION
Oligomycin A	ATP Synthase	Merck	75351	1.5 µM
2-deoxy glucose	Hexokinase	Merck	D8375	50 mM
(+)-Etomoxir (sodium salt)	carnitine palmitoyltransferase (CPT)-1	Cayman Chemical Company	11969	5 µM
Pyrrophenone	Cytosolic PLA2 (group IVA phospholipase A2 α) – reversible inhibitor	Cayman Chemical Company	13294	10 nM
MAFP (Methyl Arachidonyl Fluorophosphonate)	Cytosolic PLA2 (group IVA phospholipase A2 α) – irreversible inhibitor	MCE	HY-103334	5 µM
Adenosine quantification kit	Adenosine	Cell Biolabs	MET-5090	/

Supplemental Methods

Metabolomics

Pellets from iTreg cells (2 x 10^6) (for steady state metabolomics) or iTreg supplemented with D-Glucose-1,2-$^{13}C_2$ (10µM for 24h, for metabolic fluxes) were resuspended in 250 µl methanol/acetonitrile 1:1 containing [U-13C6]-Glucose (Merk, 389374), [U-13C5]-Glutamine (Merk, 605166), [U-13C16]-palmitic acid (Merk, 605573) 1 ng/µl as internal standards, lysed by TissueLyser at the highest frequency and then spun at 20,000g for 5 min at 4 °C. Supernatants were then passed through a regenerated cellulose filter, dried and resuspended in 100 µl of MeOH for subsequent analysis. Amino acids quantification was performed through previous derivatization. Briefly, 50 µl of 5% phenyl isothiocyanate (PITC) in 31.5% EtOH and 31.5% pyridine in water were added to 10 µl of each sample. Mixtures were then incubated with PITC solution for 20 min at RT, dried under N2 flow and suspended in 100 µl of 5 mM ammonium acetate in MeOH/H2O 1:1. Metabolomic data were performed on an API-4000 triple quadrupole mass spectrometer (AB Sciex) coupled with a HPLC system (Agilent). The identity of all metabolites was confirmed using pure standards. Quantification of different metabolites was performed with a liquid chromatography/tandem mass spectrometry (LC-MS/MS) method using a C18 column (Biocrates) for amino acids and cyano-phase LUNA column (50 mm × 4.6 mm, 5 µm; Phenomenex) for energetic metabolites, respectively. Methanolic samples were analyzed by a 10 min and 3 min run in positive (amino acids) and 5 min run in negative (all other metabolites) ion mode with a 21 multiple reaction monitoring (MRM) transitions in positive ion mode and 30 MRM transitions in negative ion mode, respectively. The mobile phases for positive ion mode analysis (amino acids) were as follows: phase A, 0.2% formic acid in water and phase B, 0.2% formic acid in acetonitrile. The gradient was T0 100%A, T5.5min. 5%A, T7min 100%A with a flow rate of 500 µl/min. The mobile phases for negative ion mode analysis (all other metabolites) were as follows: phase A, Water and B, 2 mM ammonium acetate in MeOH. The gradient was 90% B for all the analysis with a flow rate of 500 µl/min.

Liquid chromatography–electrospray ionization tandem mass spectrometry (LC–ESI–MS/MS)

iTreg (5 x 10^6) cell pellets were reconstituted in 700 µl of 1x PBS and mixed with 800 µl of 1 N hydrochloric acid:methanol (MeOH) 1:8 (v/v), 900 µl of chloroform, 200 µg/ml of the antioxidant 2,6-di-tert-butyl-4-methylphenol (BHT; Sigma–Aldrich), and 3 µl of SPLASH LIPIDOMIX Mass Spec Standard (#330707; Avanti Polar Lipids, United States, Alabaster). After vortexing and centrifugation, the lower organic fraction was collected and evaporated using a Savant Speedvac spd111v (Thermo Fisher Scientific) at room temperature, and the remaining lipid pellet was stored at −20 °C under argon. The lipid pellet was reconstituted in 100% ethanol. Lipid species were analyzed by LC–ESI–MS/MS on a Nexera X2 UHPLC system (Shimadzu, Japan, Kioto) coupled with a hybrid triple quadrupole/linear ion trap mass spectrometer (6500+ QTRAP system; AB SCIEX, The Netherlands, Nieuwerkerk aan den IJssel). Chromatographic separation was performed on an XBridge amide column (150 mm × 4.6 mm, 3.5 µm; Waters) maintained at 35 °C using mobile phase A [1 mM ammonium acetate in H2O-acetonitrile 5:95 (v/v)] and mobile phase B [1 mM ammonium acetate in H2O-acetonitrile 50:50 (v/v)] in the following gradient: (0–6 min: 0% B → 6% B; 6–10 min: 6% B → 25% B; 10–11 min: 25% B → 98% B; 11–13 min: 98% B → 100% B; 13–19 min: 100% B; 19–24 min: 0% B) at a flow rate of 0.7 ml/min, which was increased to 1.5 ml/min from 13 min onward. Sphingomyelin (SM), cholesteryl esters (CE), ceramides (CER), dihydroceramides (DCER), hexosylceramides (HCER), and lactosylceramides (LCER) were measured in positive ion mode with precursor scans of 184.1, 369.4, 264.4, 266.4, 264.4, and 264.4, respectively. Tri-, di-, and monoacylglycerides (TGs, DGs, and MGs) were measured in positive ion mode with a neutral loss scan for one of the fatty acyl moieties. Phosphatidylcholine (PC), lysophosphatidylcholine (LPC), phosphatidylethanolamine (PE), lysophosphatidylethanolamine (LPE), phosphatidylglycerol (PG), phosphatidylinositol (PI), and phosphatidylserine (PS) were measured in negative ion mode by fatty acyl fragment ions. Lipid quantification was performed by scheduled multiple reaction monitoring (MRM), and the transitions were based on neutral losses or typical product ions as described above. The instrument parameters were as follows: curtain gas = 35 psi; collision gas = 8 a.u. (medium); IonSpray voltage = 5500 V and −4500 V; temperature = 550 °C; ion source gas 1 = 50 psi; ion source gas 2 = 60 psi; declustering potential = 60 V and −80 V; entrance potential = 10 V and −10 V; collision cell exit potential = 15 V and −15 V. Peak integration was performed with MultiQuantTM software version 3.0.3.

Lipid species signals were corrected for isotopic contributions (calculated with Python Molmass 2019.1.1) and were quantified based on internal standard signals and adhered to the guidelines of the Lipidomics Standards Initiative (LSI; level 2 type quantification as defined by the LSI). Only the detectable lipid classes and fatty acyl moieties are reported in this manuscript.

RNAsequencing

iTreg (5 x 10^6) cell pellets were snap frozen in dry ice and stored at −80 °C until analysis. Total RNA from iTreg was extracted from iTreg using the Monarch total RNA miniprep kit (New England Biolabs) according to manufacturer's instructions. The RNA was subsequently quantified, and quality controlled using the Tape Station (Agilent) and Qubit fluorimeter (Invitrogen) using developer kits (ScreenTape, #5067-5576, ScreenTape Buffer, #5067-5577, ScreenTape Ladder, #5067-5578, Agilent; RNA Quantification, broad range, Thermofisher, #Q10210). Quality-checked RNA was used for library preparation with Illumina-stranded mRNA Prep ligation kit (Illumina, #20040532). Libraries concentration and fragmentation were assessed using the Qubit fluorimeter (Invitrogen) and Tape Station system (Agilent) with developer kits (Qubit dsDNA HS Assay Kit, 500 assay, Thermo fisher, # Q32854; ScreenTape D1000, #5067-5582, Reagents ScreenTape D1000, #5067-5583, Agilent) according to the developers' instruction. After libraries were pooled and denatured with the developer's kit (HT1 Buffer, Illumina, #20015892), sequencing was performed on the Illumina NextSeq 550 using the NextSeq 500/550 High Output Kit v2.5 (Illumina, #20024907). Raw sequencing data were processed employing the nf-core/rnaseq (https://zenodo.org/records/7998767) [44,45] pipeline version 3.6 with docker profile. Briefly, reads were trimmed with Trim Galore! and then aligned to the mouse reference genome (GRCm38) using STAR [46]. Gene quantification was performed via RSEM22 using RefSeq annotation. Differential expression analysis was performed with DESeq223 in R environment, taking genes with false discovery rate (FDR) < 0.05. Samples with poor RNA quality and too low read depth compared with others were excluded from RNA sequencing analysis.

RNAseq dataset has been deposited to the Sequence Read Archive (SRA) data of the NIH (https://www.ncbi.nlm.nih.gov/sra) with the Accession code PRJNA1167636.
