# Supplementary material for: SREBP1c modulates Treg immunobiology through a phospholipid-dependent adenosine pathway
Source: J Clin Invest. 2026 Jul 21;136(17):e201325. doi: 10.1172/JCI201325 (PMC13528931; doi:10.1172/JCI201325)

Figure 5G

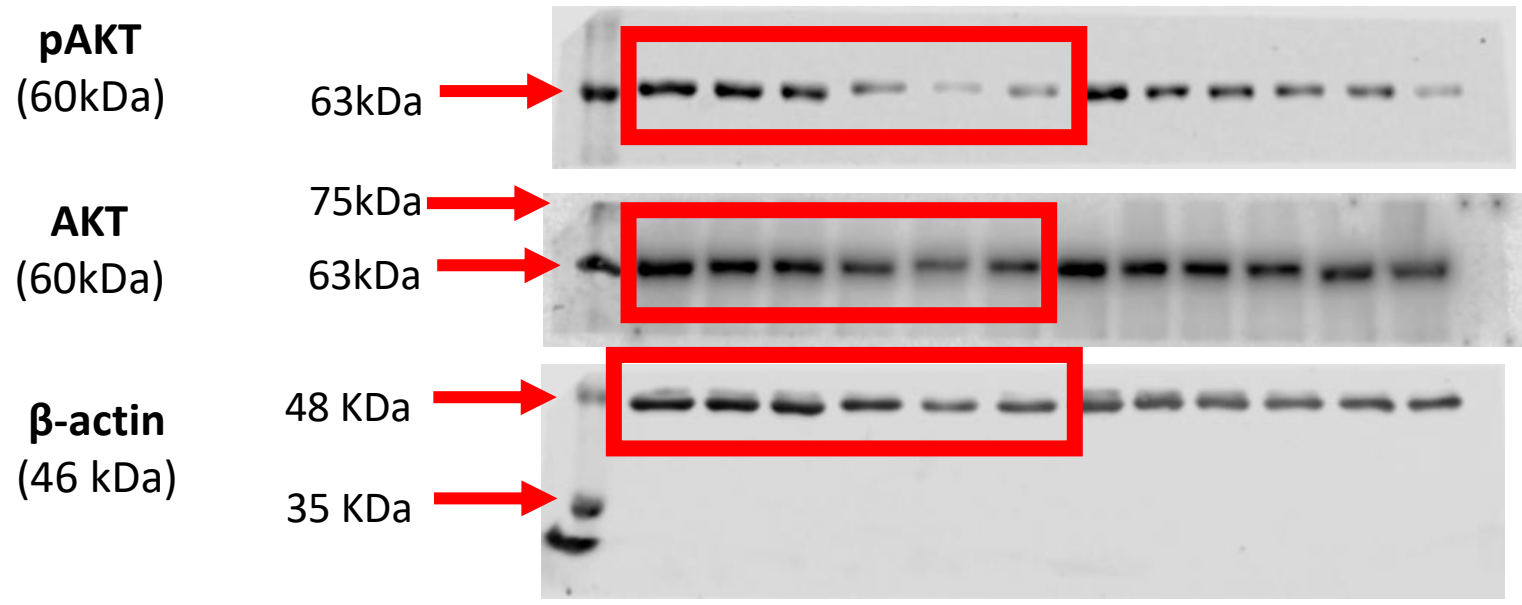

Figure 6B

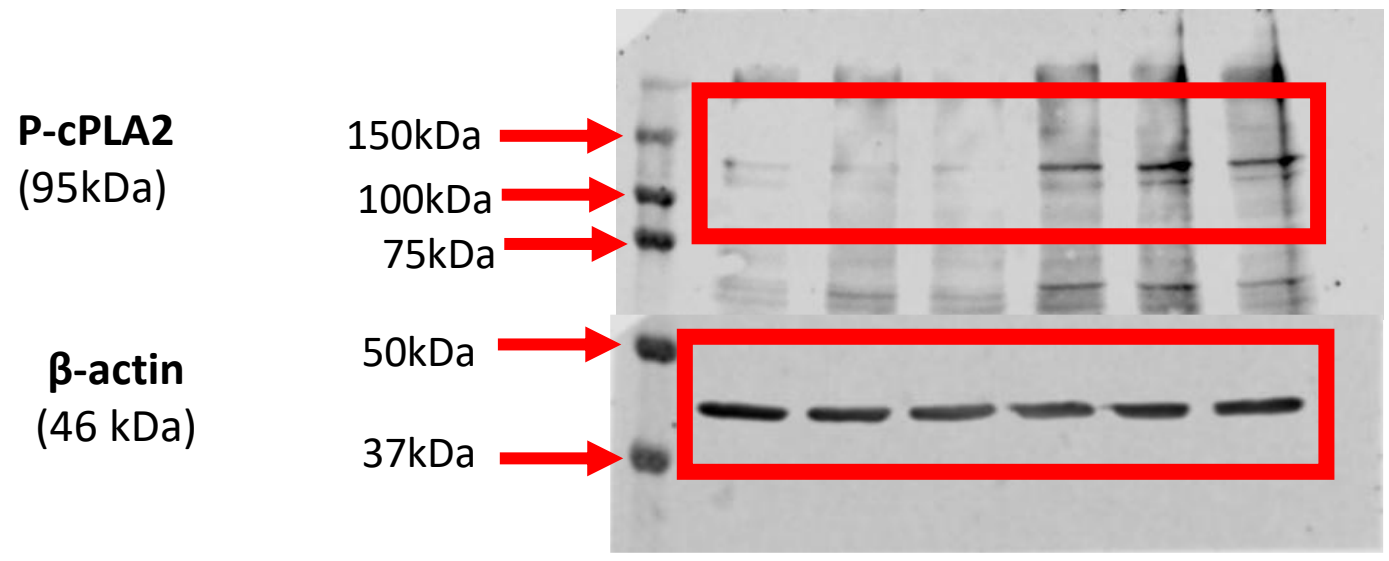

Supplemenatary Figure 6I

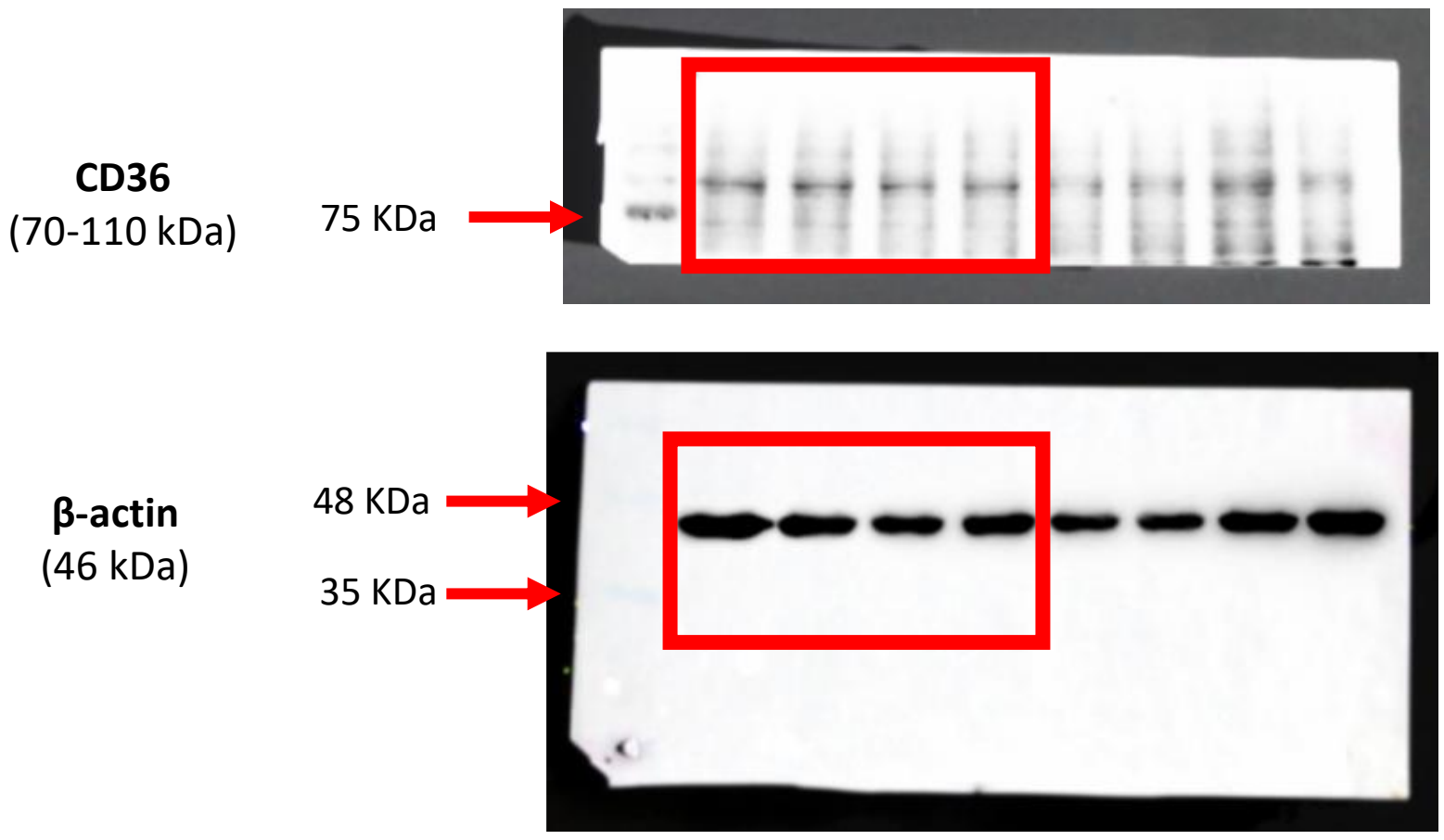

Supplemenatary Figure 9A

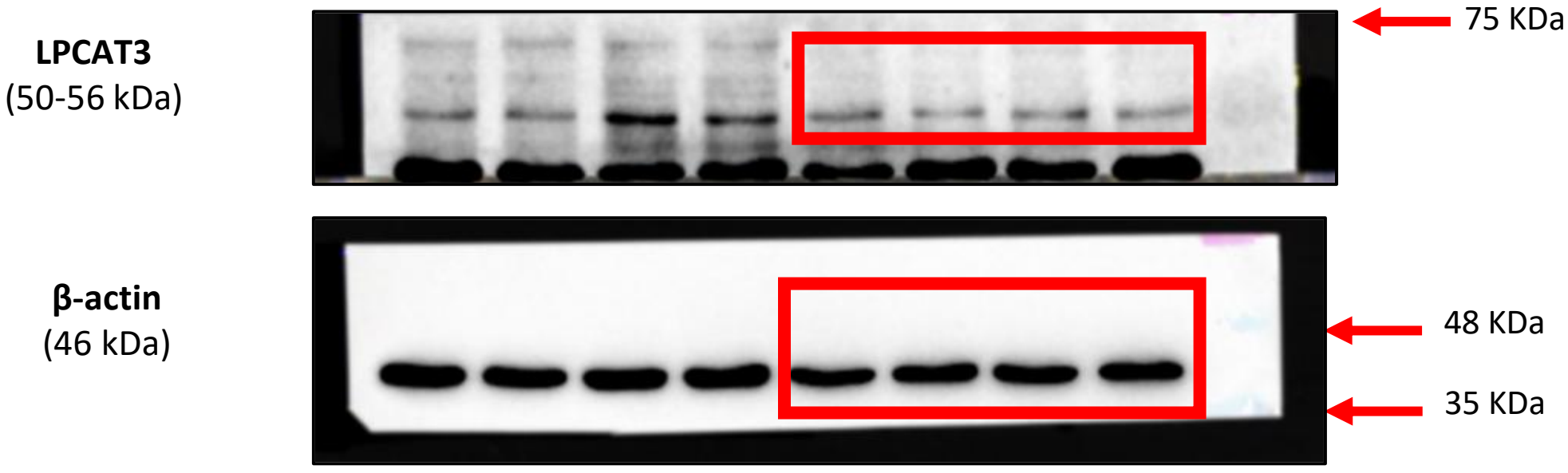

Supplement: Supplementary file 2 [file jci-136-201325-s008.pdf]
